# Quantification of miRNA-mRNA Interactions

**DOI:** 10.1371/journal.pone.0030766

**Published:** 2012-02-14

**Authors:** Ander Muniategui, Rubén Nogales-Cadenas, Miguél Vázquez, Xabier L. Aranguren, Xabier Agirre, Aernout Luttun, Felipe Prosper, Alberto Pascual-Montano, Angel Rubio

**Affiliations:** 1 Group of Bioinformatics, CEIT and TECNUN, University of Navarra, San Sebastian, Spain; 2 National Center for Biotechnology-CSIC, Madrid, Spain; 3 Structural Biology and Biocomputing Programme, Spanish National Cancer Research Centre (CNIO), Madrid, Spain; 4 Center for Molecular and Vascular Biology, Katholieke Universiteit Leuven, Leuven, Belgium; 5 Hematology Department and Area of Cell Therapy, Clínica Universidad de Navarra, Foundation for Applied Medical Research, University of Navarra, Pamplona, Spain; University of Turin, Italy

## Abstract

miRNAs are small RNA molecules (*′* 22*nt*) that interact with their corresponding target mRNAs inhibiting the translation of the mRNA into proteins and cleaving the target mRNA. This second effect diminishes the overall expression of the target mRNA. Several miRNA-mRNA relationship databases have been deployed, most of them based on sequence complementarities. However, the number of false positives in these databases is large and they do not overlap completely. Recently, it has been proposed to combine expression measurement from both miRNA and mRNA and sequence based predictions to achieve more accurate relationships. In our work, we use LASSO regression with non-positive constraints to integrate both sources of information. LASSO enforces the sparseness of the solution and the non-positive constraints restrict the search of miRNA targets to those with down-regulation effects on the mRNA expression. We named this method TaLasso (miRNA-Target LASSO).

We used TaLasso on two public datasets that have paired expression levels of human miRNAs and mRNAs. The top ranked interactions recovered by TaLasso are especially enriched (more than using any other algorithm) in experimentally validated targets. The functions of the genes with mRNA transcripts in the top-ranked interactions are meaningful. This is not the case using other algorithms.

TaLasso is available as Matlab or R code. There is also a web-based tool for human miRNAs at http://talasso.cnb.csic.es/.

## Introduction

miRNAs are small RNA molecules (*′* 22*nt*) that regulate the expression of their corresponding mRNA targets in many eukaryotes. The imperfect base pairing of the miRNAs with the 3′-untranslated region (3′-UTR) of their targets can either inhibit translation or cause mRNA cleavage [1]. Some authors have recently stated that “miRNAs predominantly act to decrease target mRNA levels” [2]. Although low translational repression with no mRNA destabilization is also possible, the overall effect is the down-regulation of both protein and mRNA concentrations. This deregulation is key in a wide range of biological processes and human diseases [3].

MiRBase [4] is the de facto standard database used to retrieve data related to miRNAs. It currently contains near 1400 human miRNAs (release 17). Considering that each miRNA has a sequence compatible with around 200 target mRNAs, the number of putative interactions is very large. Most of the computational methods developed to identify mRNA-miRNA interactions are based on sequence complementarities of miRNA and its mRNA targets. These algorithms have been used to create databases of interactions, such as miRBase [4], TargetScan [5]–[7], PicTar [8], miRanda [9] or miRGen [10]. Unfortunately, the number of false predictions using these computational methods is still high [11]. Although there are experimental tools for miRNA target validation, several involved steps must be performed to verify the authenticity of a miRNA target. Therefore, the number of experimentally-validated targets is still very low. For instance, TarBase [12] includes around 1300 experimentally-validated interactions. This number is small if compared with the number of predicted interactions in miRBase (>500000).

In recent years, different computational methods for miRNA-mRNA interaction prediction that use expression data to decipher miRNA targets have emerged. Some of them combine this information with sequence based predictions to obtain more reliable miRNA targets. Among them are GenMiR++ [13], [14], HOCTAR [15] or MAGIA [16]. The key assumption is that due to the down-regulation effect of miRNAs on its targets, their expression levels must be inversely related, i.e. if a particular miRNA expression increases, the expression of its mRNA targets must decrease.

The difference between these methods relies on the way they use this information. For example, GenMiR++ uses a Bayesian framework and states the probabilities of having an interaction between a miRNA and its putative targets. HOCTAR [15] assumes that the expression of intronic miRNAs is strongly correlated with the expression of the genes where they are located. Therefore, gene expression can be used as an estimator of intronic miRNA expression. This approach uses the advantage of a huge number of samples, for which gene expression is available, to obtain statistically significant results. In the end, HOCTAR uses simply the correlation. This measure has been also used by other authors for miRNA-mRNA target prediction [17]–[19]. On the other hand, MAGIA uses the mutual information. A possible drawback is that it is not possible to distinguish between positive and negative regulations.

Recently, other methods based on expression data analysis for miRNA-mRNA interaction prediction have been published [20], [21]. Jayaswal et al. [20] use a two step process that consists of clustering each expression data for miRNA and mRNA followed by a t-test to find significant miRNA-mRNA relationships. Li et al. [21] apply Partial Least Squares Regression to a preselected set of differentially expressed mRNAs and miRNAs.

Adding expression data to sequence based predictions has been shown to reduce false positive rate [22].

We have developed the TaLasso algorithm that combines the information of sequence databases with mRNA and miRNA expression to quantify the down-regulation between miRNAs and their putative targets. It can be considered as a filter to sequence-based databases.

Our method is based on the LASSO regression. The norm-1 penalty term of LASSO ensures the number of predicted interactions is small. In addition, we included non-positive constraints to the LASSO regression to restrict the predicted mRNA targets to those with down-regulation effects from miRNAs. TaLasso uses as initial putative targets the union of several sequence databases (miRGen [10], miRBase [4], miRanda (microRNA.org) [9], TarBase [12], miRecords [23], miRWalk [24] release of 2010). TaLasso was applied to two datasets with matched samples of mRNA and miRNA expression data.

TaLasso was validated by measuring its ability to predict experimentally-validated targets and by analysing the biological relevancy of the predicted interactions. These results were compared with other methods (GenMiR++ and Pearson correlation). We illustrate that the top interactions predicted by TaLasso are significantly enriched in validated targets and that this enrichment is larger than using GenMiR++ or Pearson correlation. Furthermore, the functions of the genes with mRNA transcripts in the top-ranked interactions are biologically sound in the context of the experiments being studied. We also show that including the non-positive constraints improves the specificity of the prediction using LASSO regression.

## Materials and Methods

Given a set of miRNA-mRNA putative interactions and given their expression values, TaLasso quantifies the down-regulation effect of each miRNA on its mRNA targets. Due to the biological complexity underlying mRNA regulation, this quantification is made by considering several simplifications.

### Assumptions

First of all, it will be assumed that the miRNAs are the only regulators of mRNA expression, considering other possible effects as part of the noise. Therefore, TaLassso –as other methods– is able to detect only the most prominent interactions.

Secondly, the model assumes that the miRNAs down-regulate their corresponding mRNA targets. Although the existence of miRNAs that act as transcription factors [1] have been shown, they will not be considered in the model.

Thirdly, TaLasso will only quantify the down-regulation effect on those miRNA-mRNA interactions from an initial set of putative miRNA-mRNA pairs (i.e. predicted from sequence based algorithms). Consequently, TaLasso will not be able to recover those interactions not included in this initial set of putative targets.

Finally, the logarithm of the mRNA expression will be assumed to be linearly dependent on the logarithm of the expression of its putative miRNA regulators.

### Mathematical model

Let vectors ***x_j_*** = [*x_j1_, x_j2_, … , x_jI_*]_1xI_ and ***z_k_*** = [*z_k1_, z_k2_, … , z_kI_*]_1xI_ be the logarithms of the expression levels of mRNA *j* and miRNA *k*, in samples *1* to *I*. Consider also that there are *J* mRNAs and *K* miRNAs. Let *c_jk_* be an indicator variable whose value is *1* if mRNA *j* is a potential target of miRNA *k* predicted in sequence databases and *0* otherwise. Then, a linear relationship between the logarithm of the expressions of each mRNA *j* and the *K* miRNAs is assumed and it is represented by the following linear model,
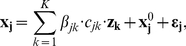
(1)where **ε_j_** is an error term and ***x_j_^0^*** = *x_j_^0^* ⋅ [*1*, *1*, … , *1*]_1xI_ is the intercept term, i.e. the expected value of the logarithm of the expression of mRNA *j* in the absence of regulation from miRNAs in samples *1* to *I*. *β_jk_* is the amount of regulation for each miRNA - mRNA pair, i.e. how much the miRNA down-regulates the target mRNA. *β_jk_* and *x_j_^0^* are unknown.

### Resolution of the linear model

In many cases, the number of miRNAs that putatively regulate an mRNA is larger than the number of samples. Therefore equation (1) is an undetermined system of equations. We propose to solve Eq (1) for each mRNA using *l_1_*-regularized least squares and adding non-positivity constraints:
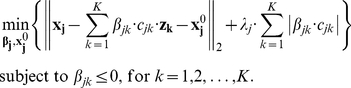
(2)


The *l_1_*-norm of the penalty term (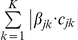) enforces the sparsity of the solution. It is adjusted with the tuning parameter *λ_j_*. The larger the value of this parameter the sparser the solution is. Non-positivity constraints are added to ensure that the solution includes only negative relationships between mRNA and miRNA expressions. This is a convex problem and thus, if an algorithm is able to find a local minimum, this minimum is guaranteed to be also global.

Equation (2) is very similar to a LASSO regression problem. The only differences are that Eq (2) includes a term that must not be penalized (***x_j_^0^***) and that there are non-positivity constraints. Some LASSO packages that include inequality restrictions are available and they can be accommodated to solve this optimization problem [25]–[27].

We have adapted two solvers for this particular problem, one for Matlab and the other one for R (http://cran.r-project.org/). TaLasso can be run in either Matlab or R. Matlab code is adapted from the *l1_ls* software (http://www.stanford.edu/~boyd/l1_ls/) [26]. Some minor changes in the initial Matlab code boosted the speed two-fold. R code was developed by using the package Rcplex (http://CRAN.R-project.org/package=Rcplex) [27]. In this case, the *l_1_*-regularized least square problem was converted into a quadratic problem by expanding the norm-2 factor. Both packages have comparable performance. Specifically, Matlab package is 30% faster.

There is no method that provides statistical significance in the context of regularized Least Squares with constraints. Although bootstrap could be an alternative, the estimation is inappropriate due to the bias and is time consuming. We have instead opted to use the statistics of multiple linear regression to assign to the solutions of TaLasso their p-vales pstatistical significance. In the software, we have included a function called *significance_beta*, in both R and Matlab codes (see texts S5 and S6 for further information).

### Selection of the *l_1_*-penalty

LASSO regression theory states [26] that the possible values of the tuning parameter *λ_j_* must lie within the interval [0, *λ_j_^max^*], being *λ_j_^max^* equal to,
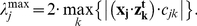
(3)


If *λ_j_* is zero, the standard minimum squares solution is obtained. If *λ_j_* is *λ_j_^max^* then the optimum solution is the null vector. The value of *λ_j_* can be selected by cross validation. It would be possible to compute *λ_j_* independently for each mRNA. However, this approach requires many additional parameters in the model and it is prone to overfitting. Instead of this, we select *λ_j_* for every mRNA as a fraction of a maximum value Λ_j_. In the following, the variable *κ* = *λ_j_*/Λ_j_, same for all mRNAs, will be used to denote these fractions.

TaLasso provides two possible ways to select Λ_j_: the global and the local methods. In the global method, Λ_j_, is selected as,
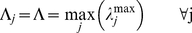
(4)And therefore,
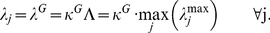
(5)


The estimation of *λ^G^* is called *global tuning parameter* in the rest of the paper and provides a single penalty term for every mRNA.

In the local method Λ_j_ is selected as,
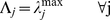
(6)and therefore,
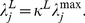
(7)


The estimation for *λ_j_^L^* in Eq (7) will be referred to as the *local tuning parameter*.

In either case we used a single *κ^L^* or for *κ^G^* for all mRNAs. Notice that, although *κ^L^* is the same for all mRNAs, *λ_j_^max^* is not and thus, *λ_j_^L^* will be different for each mRNA. In our case studies, global selection outperforms local selection. Results are included in the text S1.

The values of *κ^G^* (and *κ^L^*) were determined by testing the factors {1/10000, 1/100, 1/50, 1/20, 1/10, 1/5, 1/3, 1/2} using Leave One Out Cross Validation (LOOCV). This method performs the optimization using all samples but one and validates the results with the sample left out. This procedure is repeated for every sample. The selected value of *κ* is the one that provides the minimum square error (MSE) in the samples left out training the algorithm with the other samples, i.e,
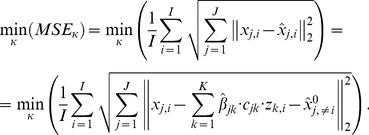
(8)where 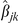 and 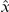 *^0^_j,_*_***≠i***_ are the estimations of the down-regulation and the intercept obtained with TaLasso for the mRNA and miRNA expression of all samples but *i*. 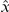 *_j,i_* is the predicted expression value of mRNA *j* in sample *i* determined by the values 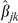. Finally, *x_j,i_* and *z_k,i_* are the expressions of mRNA *j* and miRNA *k* on sample *i*.

## Results

We have implemented the solvers for Eq (2) in R and Matlab. Putative and experimental interactions are included in the downloadable software. We have also developed a convenient web page that performs this computation. We named them “TaLasso”. Therefore, TaLasso is both the web page and the algorithm adaptations that solve the problem described in Eq. (2). The result is the amount of down-regulation of a miRNA for their targets in a particular experiment.

We run TaLasso for two real datasets and validate the results by studying the enrichment on experimentally-validated targets. We also analyze the biological relevance of the predicted interactions. In this section we present these results and include a comparison between TaLasso, GenMiR++ and correlation methods.

### Expression datasets

TaLasso has been tested with two datasets with matched miRNA and mRNA expression data. The first dataset, which will be referred as *Multi Class Cancer* (MCC) dataset, is composed of miRNA expression data from [28] and mRNA expression data from [29]. This dataset was used in GenMiR++. The second one, which will be referred to as *acute lymphoblastic Leukemia DataSet* (LDS), is publicly available in GEO (GSE14834). It corresponds to expression profiles published by [30] and is part of a case study used in MAGIA. In both datasets, the expression values were further normalized by subtracting from each mRNA or miRNA their median value.

### Enrichment on experimentally-validated targets

We compared TaLasso results with GenMiR++ and the Pearson correlation. We were not able to reproduce mutual information results obtained in MAGIA. Nevertheless, it has been stated that for normal random variables, Mutual Information (MI) can be estimated as a function of the Spearman or the Pearson correlation coefficient [31]. Therefore, if normal conditions are met, the ranking of MI results and the ranking of the absolute value of correlation are expected to be similar. We have checked that correlation performs better than the absolute value of the correlation.

The comparison of the scoring algorithms is based on measuring the enrichment of their results on experimentally-validated interactions. If the top ranked interactions of an algorithm have more experimentally-validated targets, this algorithm is expected to perform better as more predicted interactions are validated.

The union of miRBase, miRanda, miRGen, miRecords, TarBase and miRWalk were used as the initial set of interactions. Among them, TaRBase (v5), miRecords (May 2010) and miRWalk databases were used as reference of experimentally-validated targets. Whereas the interactions included in TaRBase and miRecords have been manually curated, those in MiRWalk were automatically extracted from the literature using text mining techniques. Therefore, this last database includes less reliable but more interactions than the other two.

**Figure 1:**
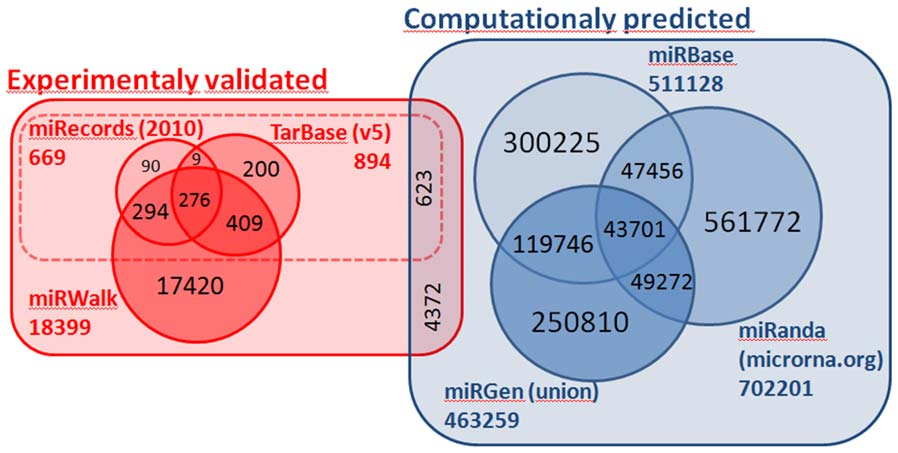
Shared interactions among the different databases of human miRNA targets that have been used as initial set of putative interactions. The overlap among the different databases is small. With reference to databases with experimentally-validated targets, the union of miRecords and TarBase includes 623 interactions that are also cited in any of the computationally predicted databases. This number rises to 4372 in case miRWalk is also considered.

Figure 1 shows the Venn diagram of the number of interactions provided by each database and the intersections among them. The number of interactions in any particular experiment is usually smaller since a particular experiment includes only a subset of the mRNAs and miRNAs in the databases. Nevertheless, the initial set of interactions comprises more than half a million putative interactions. From them, only a few (∼1000 or 10000 depending on the database) correspond to the experimentally-validated targets.

Applying TaLasso to the MCC and the LDS datasets, β, the downregulation effect of each miRNA on its targets was obtained. For both datasets we performed an enrichment analysis using the results provided by TaLasso, GenMiR++ and Correlation to mRNAs with at least one experimentally-validated miRNA target. The aim of this analysis is to compare each of these algorithms in terms of the number of experimentally-validated interactions.

The enrichment analysis was developed as follows. Firstly, the interactions were ranked according to the scores provided by each method. Secondly, taking the top-ranked *n* interactions, we counted the number of experimentally-validated targets within them. With this data, we compute the corresponding p-value using the hypergeometric distribution. The hypergeometric distribution is a discrete probability distribution that describes the probability of obtaining *p* successes (experimentally-validated interactions) when *n* elements (selected interactions) from a finite population without replacement (the union of all the putative interactions) are drawn.

These p-values help to compare the algorithms: for a given *n* (number of selected interactions) the smaller the p-value, the more enriched the solution in experimentally-validated interactions is.

We compared the algorithms using two scores: the number of experimentally validated interactions in the top-500 predicted interactions and the minimum p-value on the enrichment curves. In the second case we also provide the number of experimentally-validated targets (N_E_) and the amount of predicted interactions drawn (N_T_) on that point.

The following subsection shows the enrichment results for the union of TarBase and miRecords. The enrichment for miRWalk is included in text S1 and shows a similar trend.

#### Multi Class Cancer (MCC) expression data

The dataset used in GenMiR++ is a set of 88 paired cancer and normal samples with mRNA data from [28] and miRNA expression data from [28]. The mRNA expression data was measured with oligonucleotide microarrays Hu6800 and Hu35KsubA GeneChips (Affymetrix, Santa Clara, CA). On the other hand, the miRNA expression was obtained using bead-based flow cytometry. The dataset consists of normal and cancerous counterparts from bladder, breast, colon, kidney, lung, pancreas, prostate and uterus samples, ovary cancer, melanoma and mesothelioma samples with no normal references. We compiled the expression data from GenMiR++; that consists of 114 human miRNAs and 16063 mRNAs. Gene probe names from Hu6800 and HuK35SubA were matched with Ensembl. Correspondence was not univocal and the expression of ensemble ID genes with more than one associated probe was obtained by calculating their means. The final number of miRNAs and mRNAs after the intersection of the data with the databases included in TaLasso are 104 miRNAs and 9559 mRNAs. The total number of putative targets is 133005.

**Figure 2:**
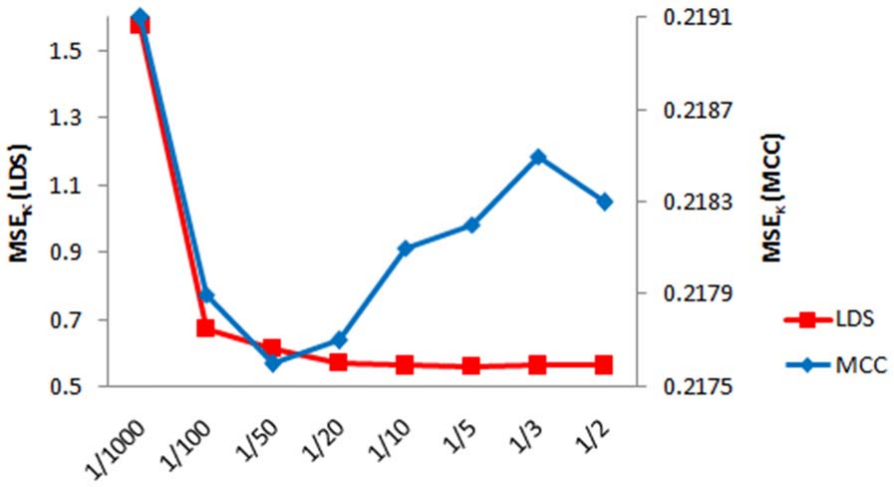
MSE errors for Cross Validation analysis for *global tuning parameters*. In the figure the LOOCV mean squared errors for different κ values of the *global tuning parameter* for MCC and LDS datasets are shown.

**Figure 3:**
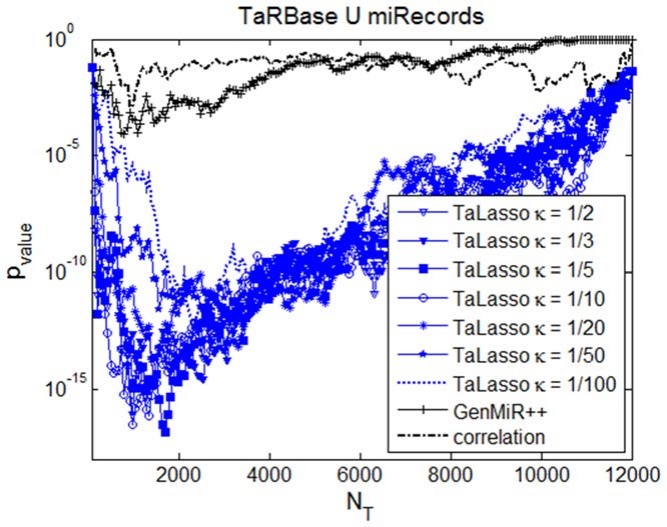
Enrichment on experimentally-validated targets for MCC dataset. For each value of the tuning factor and different number of predicted interactions, the figure shows the probability of drawing the predicted number of experimentally-validated targets by using a hypergeometric test. The figure shows TaLasso enrichment results for different *κ^G^* values (in blue), compared to the enrichment values of GenMiR++ (black crosses) and Pearson Correlation (black dashed).

For this dataset, the minimum MSE value for κ^G^ corresponds to 1/50 (figure 2) and optimal values for enrichment occur for κ^G^ about 1/10 or larger (figure 3).

The number of experimentally-validated targets within the first 500 top-ranked targets using κ^G^ higher than 1/10 is about 70% larger than using GenMiR++ or correlation (Table 1). Furthermore, the minimum p-values are lower and maximum enrichment values are higher for TaLasso than for GenMiR++ and Correlation.

**Table 1:** Maximum enrichment values on experimentally-validated targets for MCC dataset.

	TaRBase U miRecords			miRWalk		
method	N_E_/N_T_	p-value	N_E_^500^	N_E_/N_T_	p-value	N_E_^500^
***TaLasso (1/2)***	105/777	4.17E-17	67	**1761/7978**	**2.70E-52**	164
***TaLasso (1/3)***	208/2141	4.64E-16	70	1301/5591	2.20E-48	165
***TaLasso (1/5)***	**160/1413**	**3.81E-18**	65	1791/8269	1.70E-47	**172**
***TaLasso (1/10)***	113/858	1.62E-17	**74**	1441/6459	2.10E-43	170
***TaLasso (1/20)***	138/1207	6.60E-16	70	1226/5579	8.10E-33	149
***TaLasso (1/50)***	165/1689	1.16E-12	58	2420/12738	3.00E-23	106
***TaLasso (1/100)***	185/1942	3.05E-13	53	1348/6775	1.30E-17	97
***GenMiR++***	60/616	3.35E-05	46	1304/6614	1.30E-15	116
***Correlation***	63/729	6.78E-04	38	711/4004	7.90E-03	91

The table shows the maximum enrichment values (point of minimum p-value) for the union of TaRBase and miRecords, for MCC dataset. N_E_: is the number of experimentally-validated targets rescued in the point of minimum p-value and N_T_: is the total number of predicted targets in that minimum. N_E_^500^: is the amount of experimentally-validated targets in the first 500 predicted interactions.

In the case of miRWalk, the minimum p-value is obtained for a larger number of interactions than for the union of TaRBase and miRecords. We presume that this larger value is due to the larger number of interactions included in miRWalk than in the union of TaRBase and miRecords. Nevertheless, in the first 500 targets drawn, more than 1/3 of the targets are included in miRWalk.

In order to determine the effect of the addition of non-positivity constraints in target prediction, we compared enrichment results of TaLasso with and without the addition of these constraints. The results indicated that the enrichment values were much higher when non-positivity constraints were considered. Both figures of the enrichment values on the union of TaRBase and miRecords, and on miRWalk are shown in the figure S1.

#### Acute Lymphoblastic Leukemia DataSet (LDS)

The dataset used in MAGIA is a set of 19 paired T-lineage and B-lineage expression for Acute Lymphoblastic Leukemia samples (ALL) with mRNA and miRNA data from [30]. MiRNA data was obtained with miRHuman 9.0 array from LC Sciences and mRNA data with HG-U133 Plus 2.0 array from Affymetrix. The data is composed of: B-ALL with BCR/ABL, E2A/PBX1 and MLL/AF4 translocations, B-ALL without translocations, T-ALL with SIL/TAL translocations and T-ALL with no translocations.

The final common set of mRNAs and miRNAs (after intersection of the data in the arrays with the data included in TaLasso) was 16590 mRNAs and 465 miRNAs. The total set of initial putative targets was of 748305.

**Figure 4:**
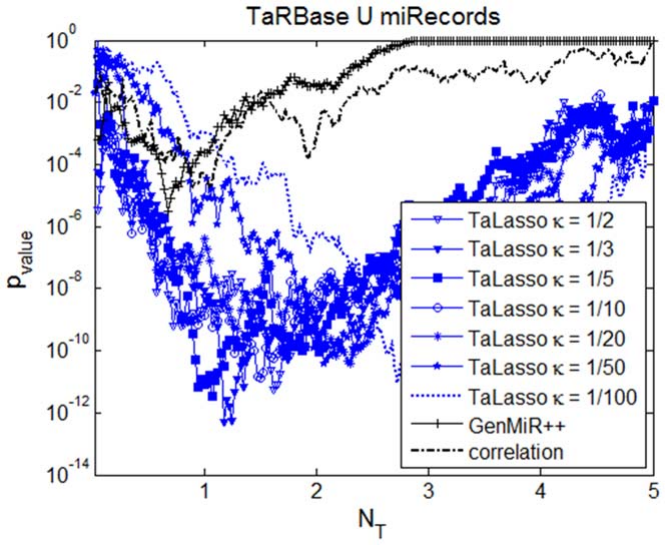
Enrichment on experimentally-validated targets for LDS dataset. For each value of the tuning factor and different number of predicted interactions, the figure shows the probability of drawing the predicted number of experimentally-validated targets by using a hypergeometric test. The figure shows TaLasso enrichment results for different *κ^G^* values (in blue), compared to the enrichment values of GenMiR++ (black crosses) and Pearson Correlation (black dashed).

For LDS dataset, minimum norms for LOOCV were found for κ^G^ equal to 1/10 or higher (figure 2). The best enrichment results were obtained for κ^G^ higher or equal to 1/5 (figure 4 and table 2).

**Table 2:** Maximum enrichment values on experimentally-validated targets for LDS dataset.

	TaRBase U miRecords			miRWalk		
method	N_E_/N_T_	p-value	N_E_^500^	N_E_/N_T_	p-value	N_E_^500^
***TaLasso (1/2)***	388/14688	4.35E-12	**23**	**4495/50098**	**2.40E-46**	83
***TaLasso (1/3)***	**313/11036**	**5.00E-13**	22	4567/51110	2.20E-45	**90**
***TaLasso (1/5)***	276/9506	1.94E-12	20	3359/36419	7.70E-41	75
***TaLasso (1/10)***	356/13234	6.80E-12	14	5559/64662	2.30E-38	42
***TaLasso (1/20)***	503/20417	1.58E-11	10	5866/69711	9.70E-30	42
***TaLasso (1/50)***	462/18408	2.00E-11	11	7368/89276	1.10E-33	39
***TaLasso (1/100)***	583/24350	3.76E-12	13	8435/103517	5.00E-37	43
***GenMiR++***	165/5936	2.80E-06	21	1908/21185	6.90E-17	50
***Correlation***	208/8040	1.51E-05	16	662/7599	8.62E-05	41

The table shows the maximum enrichment values (point of minimum p-value) for the union of TaRBase and miRecords, for MCC dataset. N_E_: is the number of experimentally-validated targets rescued in the point of minimum p-value and N_T_: is the total number of predicted targets in that minimum. N_E_^500^: is the amount of experimentally-validated targets in the first 500 predicted interactions.

The number of experimentally-validated targets in the first 500 interactions for LDS dataset is smaller than for MCC but still higher than using GenMiR++ or correlation. In the case of the miRWalk database the difference is more apparent.

Once again, results with non-positivity constraints performed better than TaLasso without these constraints. These results are shown in the figure S2.

### KEGG pathway enrichment

In order to analyze the biological relevance of the results, KEGG pathway enrichment analysis and a literature review of the experimentally-validated targets predicted were carried out. We selected κ^G^ = 1/5 and 1/10 in MCC and κ^G^ = 1/2 and 1/3 in LDS TaLasso results for their comparison with the results of GenMiR++ and Pearson Correlation.

GeneCoDis 2.0 web-based tool [32], [33] was used for KEGG pathway enrichment analysis. We measured the statistical significance using the hypergeometric distribution. Afterwards, the p-values were corrected by FDR. Enrichment of KEGG pathways of the first 200 different genes within the top-ranked interactions was then computed.

#### Results for Multi Class Cancer (MCC) data

A large amount of experimentally-validated interactions within the top 500 predicted by TaLasso corresponded to miR-1. This did not occur to the top 500 interactions of GenMiR++ and Pearson Correlation. However, GenMiR++ predicted two of those miR-1 targets in the top 500 interactions.

Although miR-1 is muscle specific, we found relationships between miR-1 and cancer. In MCC data, miR-1 was found to be differentially expressed between normal and tumoral colon samples as was also observed in [34]. We found that miR-1 expression was also down-regulated in normal prostate and uterus tumors in MCC data. Furthermore, some of the targets predicted by TaLasso for miR-1 were related to the cancers in MCC data: for instance, *MCM7*, related to prostate cancer progression [35] and *TAGLN2*, a colorectal cancer biomarker [35].

No KEGG pathway enrichment related to cancer was observed either for TaLasso or GenMiR++ and Correlation. We associated the lack of KEGG enrichment to the large number of cancer types in MCC data.

Contrary to KEGG pathway analysis, the literature showed several associations with cancer for the experimentally-validated targets. The number of experimentally-validated interactions associated to cancers in MCC data was higher for TaLasso than for GenMiR++ and Pearson Correlation (table 3, corresponding references are included in text S3).

**Table 3:** Predicted experimentally-validated targets and the cancer to which they have been related in the literature: results for MCC dataset.

Interactions		Database			Method				Associated to								
*Gene*	*miRNA*	*TaRBase*	*miRecords*	*miRWalk*	*TaLasso (1/5)*	*TaLasso (1/10)*	*GenMiR++*	*Correlation*	*OV*	*BC*	*EC*	*CC*	*LC*	*BrC*	*NC*	*PC*	*GC*
EEF1A2	let-7f		**X**	**X**	**X**	**X**			**X**								
FSCN1	miR-133a			**X**	**X**	**X**				**X**	**X**						
FSCN1	miR-145			**X**	**X**	**X**				**X**	**X**	**X**					
BRAF	miR-192			**X**		**X**						**X**					
CCND1	miR-194			**X**		**X**											
HOXD10	miR-200c			**X**		**X**				**X**		**X**	**X**	**X**			
CDKN2A	miR-99a			**X**		**X**							**X**				
TFF1	let-7f		**X**	**X**	**X**				**X**								
CEACAM5	miR-143/145			**X**	**X**							**X**					
KRT14	miR-143			**X**	**X**						**X**						
KRT7	miR-145/195			**X**	**X**					**X**							
INS	miR-27a			**X**	**X**				**X**					**X**			
IGFBP6	miR-27a			**X**	**X**				**X**					**X**			
PIGR	miR-125b		**X**	**X**			**X**									**X**	
BRAF	miR-145			**X**			**X**					**X**					
PLK1	miR-100			**X**				**X**							**X**		
BAK1	miR-125b		**X**					**X**						**X**			
E2F1	miR-195			**X**				**X**									**X**
TWIST1	miR-141			**X**				**X**						**X**			
TWIST1	miR-200c			**X**				**X**		**X**				**X**			

OV: Ovarian Cancer, BlC: Bladder Cancer; EC: Esophageal Cancer; CC: Colon/Colorectal Cancer; LC: Lung Cancer; BrC: Breast Cancer, NC: Nasopharingeal Cancer, PC: Prostate Cancer; GC: Gastric Cancer.

The experimentally-validated targets included in the top 500 targets predicted were selected and their literature references included on TaRBase, miRecords and miRWalk were analyzed in search of biological relevancy. In the table only those interactions with a literature reference related with MCC environment have been included. This was made for the predictions of TaLasso, GenMiR++ and Pearson Correlation.

#### Results for Leukaemia DataSet (LDS)

**Figure 5:**
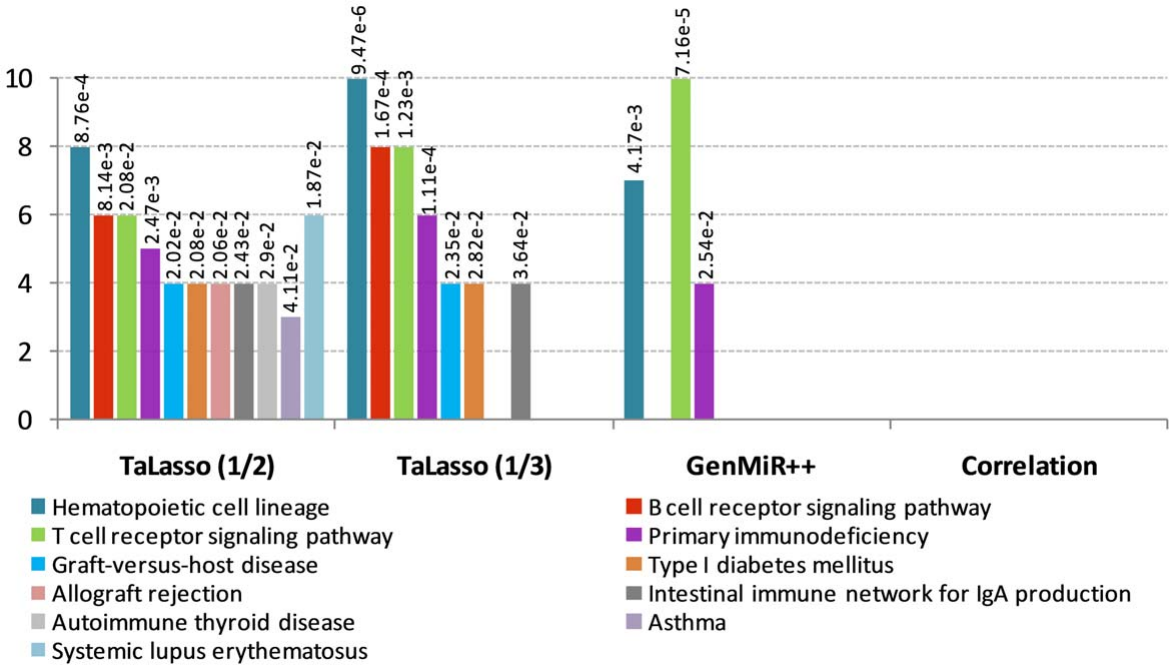
KEGG pathway enrichment results for LDS dataset. Enrichment analysis on KEGG pathways of the 200 top-ranked genes. The figure shows the results for TaLasso, GenMiR++ and Pearson Correlation. In the figure, the x-axis indicates the number of mRNAs on each enriched pathway. The associated p-value is also shown. The list of genes on each enriched KEGG pathway is included in text S2.

In LDS, several significant KEGG pathways were found for TaLasso and GenMiR++ results (figure 5). TaLasso pathways showed to be enriched in genes associated to T- and B- cells, while GenMiR++ results were only enriched on pathways associated to T- cells. Furthermore, the number of genes associated to hematopoietic cell lineage and primary immunodeficiency was higher in TaLasso. Other diverse enriched KEGG pathways associated to immune diseases were also found by TaLasso results.

Concerning literature review of the experimentally-validated targets within the top 500 interactions (table 4, corresponding references are included in text S4), results showed a similar number of predicted interactions for GenMiR++ and TaLasso.

**Table 4:** Predicted experimentally-validated targets and the cancer to which they have been related in the literature: results for LDS dataset.

Interactions		Exp. Val. Database			Method				Associated to			
*gene*	*miRNA*	*TaRBase*	*miRecords*	*miRWalk*	*TaLasso (1/2)*	*TaLasso (1/3)*	*GenMiR++*	*Correlation*	*CLL*	*ALL*	*AML*	*IC, IR, HSC*
GPR160	miR-125b		**X**	**X**		**X**						**X**
FLT3	miR-148a			**X**	**X**	**X**					**X**	
BAALC	miR-148a			**X**	**X**	**X**					**X**	
TCL1A	miR-15a, miR-16			**X**		**X**			**X**			
TCL1A	miR-181b, miR-29b			**X**		**X**			**X**			
TCL1A	miR-34b			**X**		**X**			**X**			
CD19	miR-29c			**X**		**X**			**X**	**X**		
HLA-DPA1	miR-181a, miR-223			**X**		**X**						**X**
KIT	miR-221, miR-222	**X**	**X**	**X**			**X**				**X**	**X**
ZAP70	miR-155			**X**			**X**		**X**			
ZAP70	miR-16			**X**			**X**		**X**			
ZAP70	miR-181a			**X**			**X**		**X**			
PLK2	miR-126			**X**			**X**			**X**		
ETS1	miR-155			**X**			**X**					**X**
HOXA9	miR-126			**X**			**X**					**X**
CCNA1	let-7b	**X**		**X**		**X**	**X**	**X**				

CLL: Chronic Lymphoblastic Leukaemia, ALL: Acute Lymphoblastic Leukaemia, AML: Acute Myeloid Leukaemia, IC: Immunce Cells, IR: Immune Response, HSC: Haematopoietic SC.

The experimentally-validated targets included in the top 500 targets predicted were selected and their literature references included on TaRBase, miRecords and miRWalk were analyzed in search of biological relevancy. In the table only those interactions with a literature reference related with LDS environment have been included. This was made for the predictions of TaLasso, GenMiR++ and Pearson Correlation.

Among the list of interactions of table 4, *BAALC*, *FTL3*, *CCNA1* and *CD19* are of particular interest.

ALL leukemia with *MLL* translocation –*called MLL– is characterized to have a high expression of HOXA9*, *FLT3*, *CCNA1 and MEIS1* and a low expression of *MME*. This expression profile allows MLL to be differentiated from other ALL types [36]. For instance, *HOXA9* and *CCNA1* were found to be highly expressed on LDS data in samples with a *MLL/AF4* translocation.

*BAALC* seems to be relevant, not only for differentiating acute myeloid leukemia [37] but also for prognosis in adult T-ALL; high *BAALC* and *ERG* expressions are associated with a high risk of relapse and inferior survival [38].

Furthermore, *CD19* and miR-29c were not predicted by GenMiR++ and were found to be related to Chronic Lymphocytic Leukemia and Acute Lymphocytic leukemia in the literature review.

*MME*, *FLT3* and *CD19* genes were also found on KEGG pathway enrichment analysis associated to hematopoietic cell lineage pathway (the list of mRNAs on each KEGG pathway can be seen in text S2; *MME* and *FLT3* appeared in TaLasso results (κ^G^ = 1/2 and 1/3) and the last one in GenMiR++ results).

### Implementation

There is a convenient web-based tool available at http://talasso.cnb.csic.es/, which implements TaLasso method, Pearson Correlation and GenMiR++ algorithms.

In order to run TaLasso in the web page, two tab- separated text files must be uploaded; one with mRNA expression data and the other with miRNA expression data. Afterwards, the user can select the databases for scoring and enrichment analysis, the units of miRNA data (ΔΔCts or expression values) and the algorithm to score the interactions.

The resulting targets are sorted by computed score and it is also shown if they are included in the given experimental validated databases. For KEGG pathway enrichment analysis of the results, a link to GeneCoDis 2.0 is added.

Matlab or R codes for TaLasso are also downloadable from the web page. A larger description of the tool is included in the texts S5.

## Discussion

We have shown that TaLasso is an effective algorithm to predict mRNA-miRNA interactions. We have compared TaLasso with GenMiR++ and correlation. Two validation methods have been used: enrichment of the predictions on experimentally- validated targets and biological interpretation of the top-ranked interactions. Results have shown that top interactions predicted by TaLasso are: significantly more enriched on validated targets and biologically more meaningful; more than the results of GenMiR++ and correlation.

Even though, in the case of MCC, Talasso showed no enriched pathways, none of the other methods showed any enrichment of pathways either. Probably, the lack of enriched pathways is more related with the data themselves than with the scoring method.

The main assumptions in TaLasso are the down-regulation effect of miRNAs on mRNA targets and the enforced sparseness of the solution. One of the key assumptions in TaLasso is that miRNAs only down-regulate their corresponding targets. This assumption has been included in LASSO by adding non-positivity constraints. However, we have also solved equation (2) without these constraints and showed that the obtained results are clearly inferior. One surprising result of our analysis is the poor performance of the correlation coefficient. We hypothesize that, if an mRNA is being regularized by two miRNAs, each acting (expressed) in different conditions, the correlation between these miRNAs and the mRNAs will be poor. In the contrary, TaLasso and GenMiR++ will assign high scores to both miRNAs since the combination of their expressions explain that of the mRNA.

TaLasso assumes that the only regulators of mRNA expression are the miRNAs. This means that in case large numbers of conditions are considered this assumption is less feasible and thus, TaLasso will be unable to retrieve subtle miRNA-mRNA interactions. Even though results for MCC (that include many different conditions) show enrichment in experimentally validated interactions; GenMiR++ and Pearson correlation are also able to recover a significant number of validated interactions.

We compared two different selections of the penalty term: using the same penalty (global selection) and different penalties (local selection) for all the mRNAs. Best results were obtained using the global selection. An intuitive explanation of this could be the following, adjusting the *l_1_*-regularized least-squared problem for each of the mRNAs, retrieves the most interesting miRNAs regulators for each mRNA. On the contrary, a global regularization parameter makes the solutions obtained for each of the mRNAs comparable between them. Intuitively, a general regularization parameter makes TaLasso select the best interactions from the whole set of putative interactions.

In conclusion, TaLasso solves an *l_1_*-regularized least-squared convex problem with non-positive restrictions to score putative targets by using the expression data of mRNAs and miRNAs. TaLasso results for MCC and LDS datasets show that the enrichment in experimentally validated targets is larger than using other known methods. Furthermore, the targets and interactions obtained by TaLasso are biologically more meaningful.

TaLasso is publicly available as Matlab or R programs. There is also a web-based tool (http://talasso.cnb.csic.es/).

Just before the submission of this manuscript, we became aware of a related works by Lu et al. [39] (“A LASSO regression model for the construction of microRNA target regulatory networks”). These authors consider the use of LASSO regression for deciphering miRNA-mRNA interactions by using expression data, sequence based predictions, RISC availability and miRNA co-regulation. The main difference between this work and this manuscript is that they do not include non-positivity constraints in their regularized least squares. Here we have shown that the addition of non-positivity constraints is crucial for miRNA-mRNA interaction search. We also provide a convenient web-page to test our model making it more readily available.

## Supporting Information

Figure S1 **Comparison of enrichment results on experimentally-validated targets with and without non-positivity restrictions: MCC dataset.** In the figure, the best results obtained with TaLasso with (blue) and without (red) the addition of non-positivity constraints, as well as the results of GenMiR++ and Pearson Correlation are shown.
(TIF)

Figure S2 **Comparison of enrichment results on experimentally-validated targets with and without non-positivity restrictions: LDS dataset.** In the figure, the best results obtained with TaLasso with (blue) and without (red) the addition of non-positivity constraints, as well as the results of GenMiR++ and Pearson Correlation are shown.
(TIF)

Text S1 **Comparison of the results for TaLasso using global and local tuning parameters with non-positivity constraints.**
(DOC)

Text S2 **List of the genes enriched in KEGG pathways for the LDS dataset.**
(DOC)

Text S3 **Reference articles for the experimentally-validated targets on the top 500 interactions for MCC dataset.**
(DOC)

Text S4 **Reference articles for the experimentally-validated targets on the top 500 interactions for LDS dataset.**
(DOC)

Text S5 **Implementation guidelines for the software and the web-page for running TaLasso.**
(DOC)

Text S6 **Statistical significance of the results obtained by TaLasso.**
(DOC)
