# Supplementary figures and images for: Quantification of miRNA-mRNA Interactions
Source: PLoS One. 2012 Feb 14;7(2):e30766. doi: 10.1371/journal.pone.0030766 (PMC3279346; doi:10.1371/journal.pone.0030766)

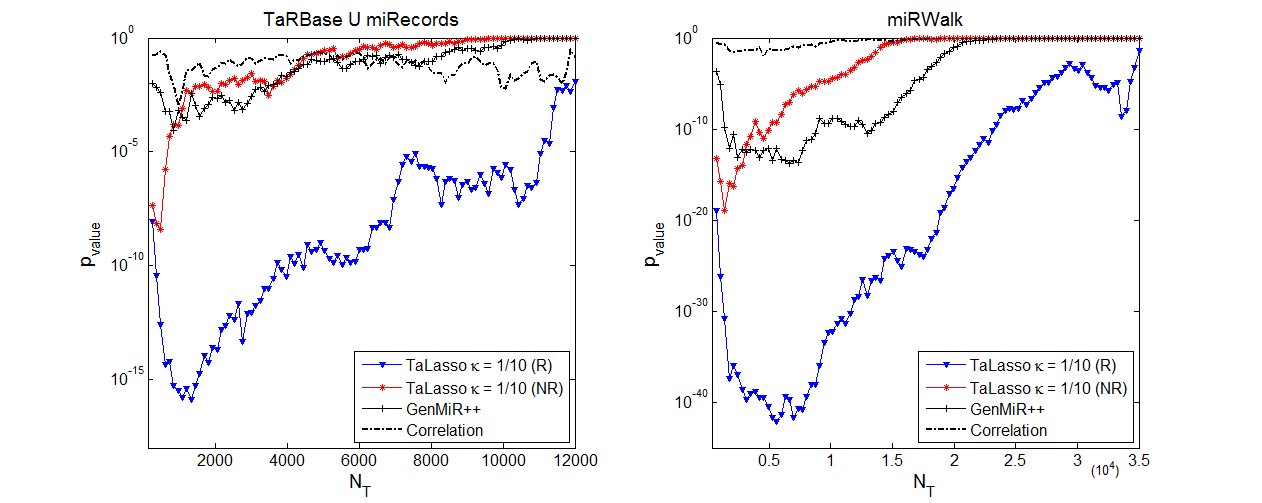

Supplement: Figure S1 — Comparison of enrichment results on experimentally-validated targets with and without non-positivity restrictions: MCC dataset. In the figure, the best results obtained with TaLasso with (blue) and without (red) the addition of non-positivity constraints, as well as the results of GenMiR++ and Pearson Correlation are shown. (TIF) [file pone.0030766.s001.tif]

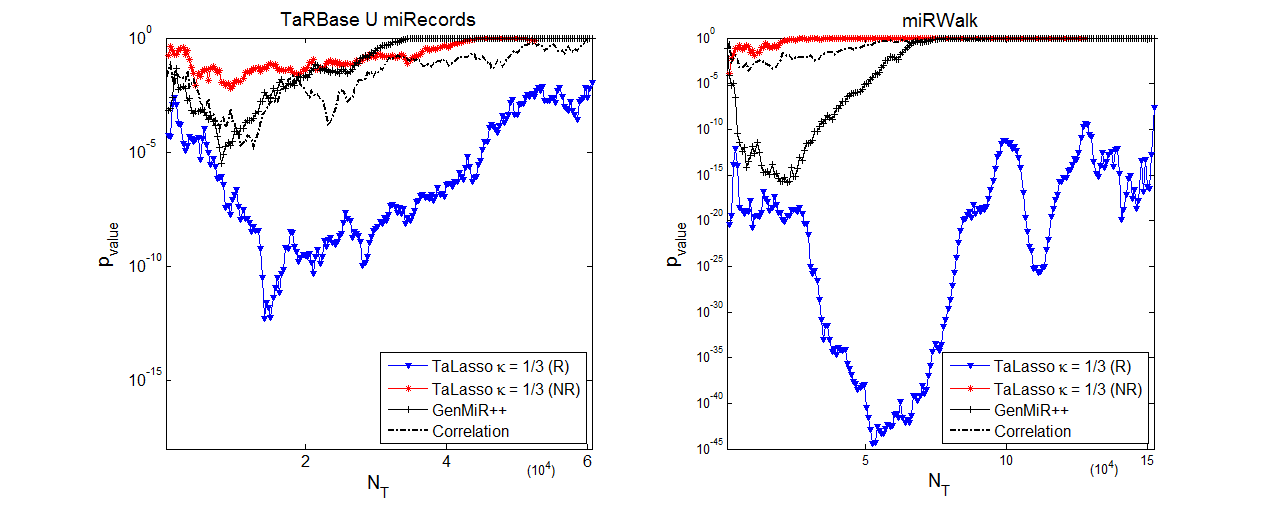

Supplement: Figure S2 — Comparison of enrichment results on experimentally-validated targets with and without non-positivity restrictions: LDS dataset. In the figure, the best results obtained with TaLasso with (blue) and without (red) the addition of non-positivity constraints, as well as the results of GenMiR++ and Pearson Correlation are shown. (TIF) [file pone.0030766.s002.tif]
