# Supplementary material for: Quantification of miRNA-mRNA Interactions
Source: PLoS One. 2012 Feb 14;7(2):e30766. doi: 10.1371/journal.pone.0030766 (PMC3279346; doi:10.1371/journal.pone.0030766)
Supplement: Text S1 — Comparison of the results for TaLasso using global and local tuning parameters with non-positivity constraints. (DOC) [file pone.0030766.s003.doc]

## Comparing *global* and *local tuning parameter* results for TaLasso with non-positivity constraints

## LOOCV results:

Here LOOCV results for different κ types (global and local tuning parameters) and values (1/2, 1/3, 1/5, 1/10, 1/20, 1/50 and 1/100), for *MCC* and *LDS* datasets for TaLasso with non-positivity constraints are included.

a)

b)

c)

**Figure 1:** **MSE errors for Cross Validation analysis.** In the figure the LOOCV mean squared errors for different κ values for *MCC* (figure a) and *LDS* (figures b and c) datasets are shown.

1. **Enrichement on experimentally validated targets:**

Figure 1 and 2 show the enrichment values on the union of TaRBase and miRecords, and on miRWalk, for TaLasso with non-negativity constraints for *global* and *local tuning parameters*. Each of the figures corresponds to *MCC* and *LDS* datasets respectively. The amount of experimentally validated targets, the number of predicted putative targets and the enrichment values on the maximum enrichment points for both dataset for *local tuning parameters* are included on tables 1 and 2. For maximum enrichment results for *global tuning parameters* see the tables 1 and 2 included on the article.

Both figures show that results for local tuning parameters are more sensitive to the values of the tuning parameter and that best results are obtained for *global tuning parameters*.


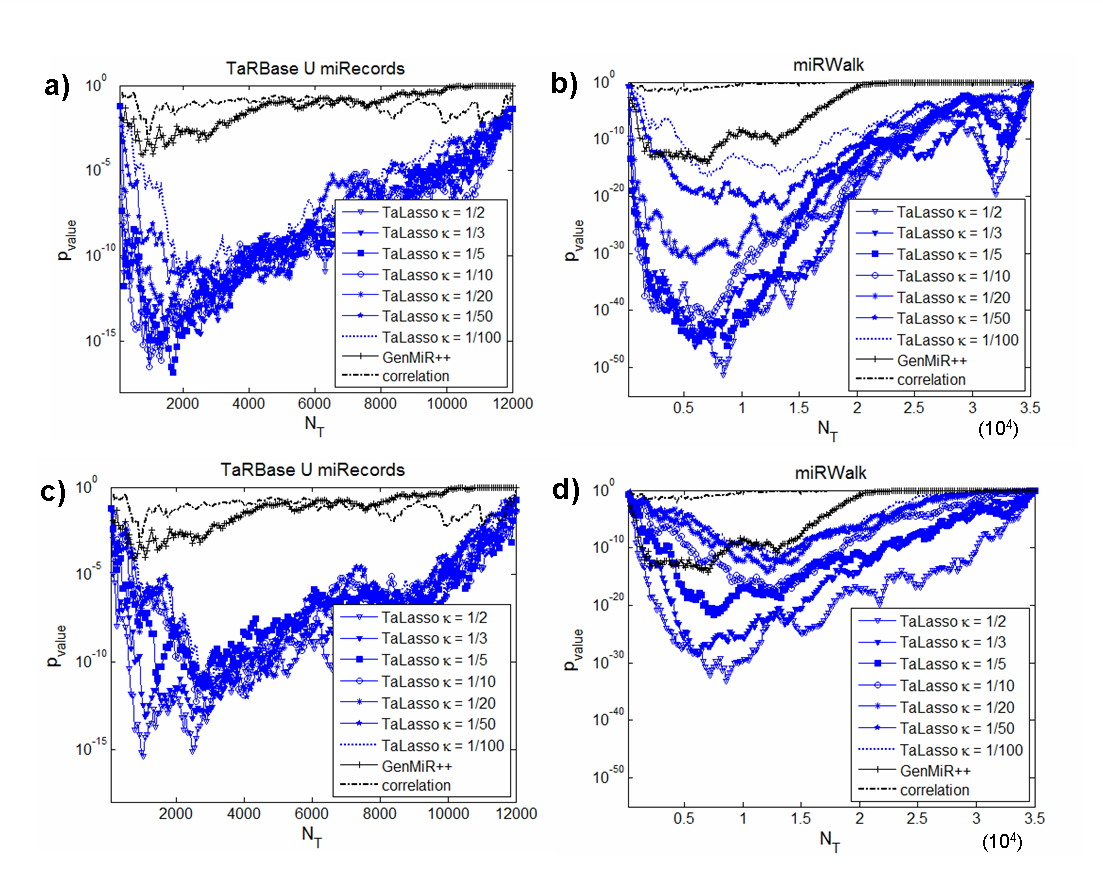


**Figure 2: Enrichment on experimentally validated targets for MCC dataset for both *local and global tunint parameters*.**

For each value of the tuning factor and different number of predicted interactions, the figure shows the probability of drawing the predicted amount of experimentally validated targets by using a hypergeometric test. The figure shows TaLasso enrichment results for different *κ^G^* and *κ^L^* values (in blue), compared to the enrichment values of GenMiR++ (black crosses) and Pearson Correlation (black dashed). Figures above (a and b) correspond to TaLasso results for *global tuning parameters* and figures below (c and d) to TaLasso results for *local tuning parameters*.

**Table 1:** **Maximum enrichment values on experimentally validated targets for MCC dataset using local tuning parameters.**

|  | | **TaRBase U miRecords** | | | **miRWalk** | | |
| --- | --- | --- | --- | --- | --- | --- | --- |
|  |  | **N_E_ / N_T_ (ρ_hyp_)** | | **N_E_^500^** | **N_exp_ / N_total_ (ρ_hyp_)** | | **N_E_^500^** |
|  |  |  |  |  |  |  |  |
| **TaLasso** | 1/2 | **108 / 825** | **(1.45e-16)** | **67** | **1692 / 8104** | **(2.88e-34)** | **122** |
|  | 1/3 | 114 / 960 | (2.56e-14) | 63 | 1255 / 5806 | (2,12e-30) | 111 |
|  | 1/5 | 219 / 2483 | (2.76e-12) | 56 | 1436 / 7068 | (1,22e-22) | 112 |
|  | 1/10 | 231 / 2619 | (4.4e-13) | 55 | 2262 / 12032 | (1,75e-18) | 113 |
|  | 1/20 | 216 / 2448 | (4.14e-12) | 45 | 2136 / 11448 | (1,83e-15) | 104 |
|  | 1/50 | 290 / 3547 | (9.2e-13) | 48 | 2151 / 11651 | (1,72e-13) | 101 |
|  | 1/100 | 227 / 2571 | (7.29e-13) | 48 | 2222 / 12140 | (2,08e-12) | 104 |
| **GenMiR++** | | 60 / 616 | (3.35e-5) | 46 | 1304 / 6614 | (1,27e-15) | 116 |
| **Correlation** | | 63 / 729 | (6.8e-4) | 38 | 711 / 4004 | (7.9e-3) | 91 |

The table shows the maximum enrichment values (point of minimum p-value) for the union of TaRBase and miRecords, for MCC dataset. N_E_: is the number of experimentally validated targets rescued in the point of minimum p-value and N_T_: is the total number of predicted targets in that minimum. N_E_^500^: is the amount of experimentally validated targets in the first 500 predicted interactions.


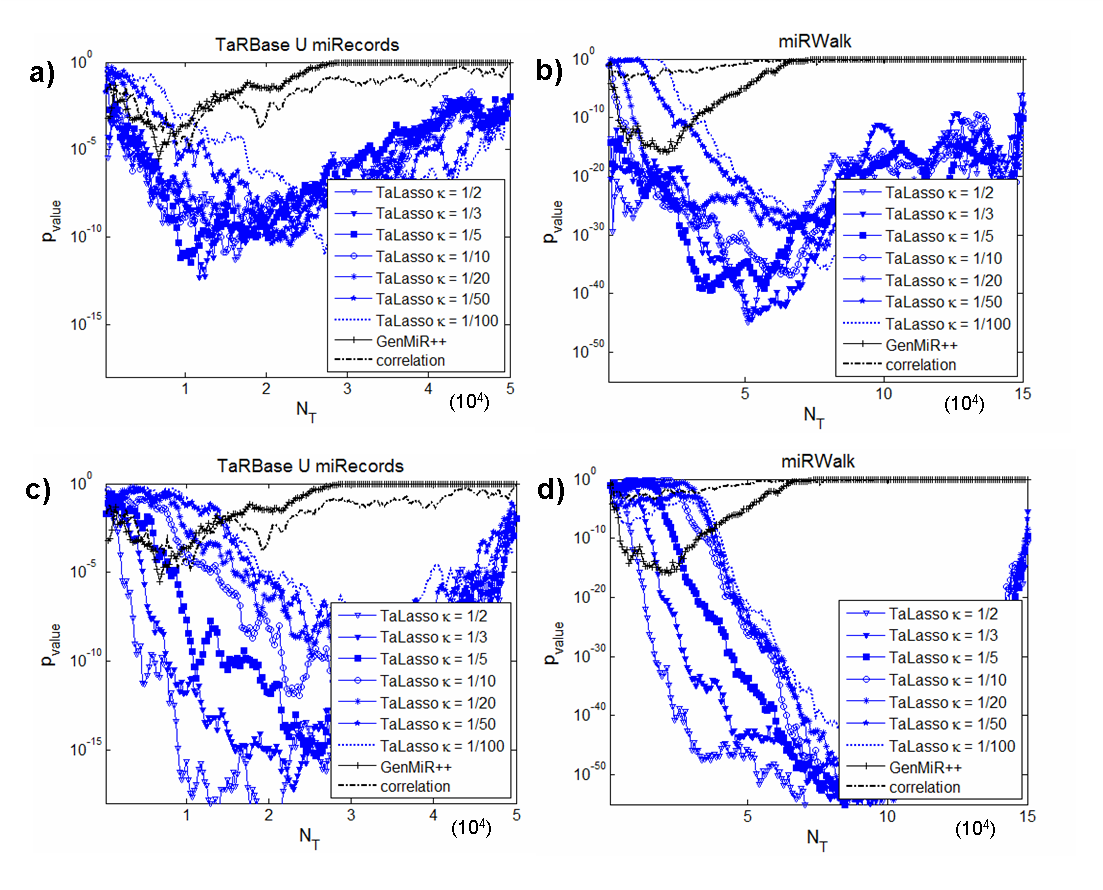


**Figure 3: Enrichment on experimentally validated targets for LDS dataset for both *local and global tunint parameters*.**

For each value of the tuning factor and different number of predicted interactions, the figure shows the probability of drawing the predicted amount of experimentally validated targets by using a hypergeometric test. The figure shows TaLasso enrichment results for different *κ^G^* and *κ^L^* values (in blue), compared to the enrichment values of GenMiR++ (black crosses) and Pearson Correlation (black dashed). Figures above (a and b) correspond to TaLasso results for *global tuning parameters* and figures below (c and d) to TaLasso results for *local tuning parameters*.

**Table 2:** **Maximum enrichment values on experimentally validated targets for LDS dataset using local tuning parameters.**

|  | | **TaRBase U miRecords** | | | **miRWalk** | | |
| --- | --- | --- | --- | --- | --- | --- | --- |
|  |  | **N_E_ / N_T_ (ρ_hyp_)** | | **N_E_^500^** | **N_exp_ / N_total_ (ρ_hyp_)** | | **N_E_^500^** |
|  |  |  |  |  |  |  |  |
| **TaLasso** | 1/2 | **483 / 17589** | **(2.53e-020)** | 14 | **7234 / 84119** | **(2.28e-65)** | 48 |
|  | 1/3 | 530 / 20318 | (3.85e-018) | 13 | 7117 / 83230 | (6,22e-58) | 48 |
|  | 1/5 | 576 / 23078 | (3.97e-016) | 12 | 7077 / 82766 | (3,81e-57) | 49 |
|  | 1/10 | 612 / 25336 | (1.92e-014) | 17 | 7306 / 86069 | (4,52e-55) | 48 |
|  | 1/20 | 648 / 27756 | (2.33e-012) | 15 | 8324 / 100083 | (2,92e-55) | **51** |
|  | 1/50 | 714 / 31790 | (8.07e-011) | 15 | 8283 / 99666 | (1,28e-53) | 50 |
|  | 1/100 | 652 / 28776 | (2.82e-009) | 13 | 7526 / 89836 | (1,69e-47) | **51** |
| **GenMiR++** | | 165 / 5936 | (2.79e-006) | **21** | 1908 / 21185 | (6,89e-17) | 50 |
| **Correlation** | | 208 / 8040 | (1.51e-5) | 16 | 662 / 7599 | (8.62e-5) | 41 |

The table shows the maximum enrichment values (point of minimum p-value) for the union of TaRBase and miRecords, for MCC dataset. N_E_: is the number of experimentally validated targets rescued in the point of minimum p-value and N_T_: is the total number of predicted targets in that minimum. N_E_^500^: is the amount of experimentally validated targets in the first 500 predicted interactions.
