# Supplementary material for: Quantification of miRNA-mRNA Interactions
Source: PLoS One. 2012 Feb 14;7(2):e30766. doi: 10.1371/journal.pone.0030766 (PMC3279346; doi:10.1371/journal.pone.0030766)
Supplement: Text S2 — List of the genes enriched in KEGG pathways for the LDS dataset. (DOC) [file pone.0030766.s004.doc]

## Genes with enriched KEGG pathways (LDS dataset)

KEGG pathway enrichment of the genes with mRNA transcripts in the 200 top-ranked interactions is shown in the following table. This table extends the information of figure 5 included in the article. In the following table, the column “also in” indicates if the corresponding gene with a particular enriched KEGG pathway in TaLasso (GenMiR++) results has been also enriched in GenMiR++ (TaLasso) results. No enriched KEGG pathway was found for Pearson Correlation results.

|  |  |  | **Enriched KEGG pathway** | | | | | | | | | | |  | **also in** | | |
| --- | --- | --- | --- | --- | --- | --- | --- | --- | --- | --- | --- | --- | --- | --- | --- | --- | --- |
|  |  |  | Hematopoietic cell | B- cell receptor | T- cell receptor | Primary immunodeficiency | Graft vs host disease | Type I diabetes mellitus | Allograft rejection | Intestinal immune network for IgA production | Autoimmune thyroid disease | Asthma | Systemic lupus erythematosus |  | TaLasso (1/2) | TaLasso (1/3) | GenMiR++ |
| TaLasso (1/2) | BLNK | B-cell linker |  |  |  |  |  |  |  |  |  |  |  |  |  |  |  |
|  | CD19 | CD19 molecule |  |  |  |  |  |  |  |  |  |  |  |  |  |  |  |
|  | CD3D | CD3d molecule, delta (CD3-TCR complex) |  |  |  |  |  |  |  |  |  |  |  |  |  |  |  |
|  | CD3G | CD3g molecule, gamma (CD3-TCR complex) |  |  |  |  |  |  |  |  |  |  |  |  |  |  |  |
|  | CD59 | CD59 molecule, complement regulatory protein |  |  |  |  |  |  |  |  |  |  |  |  |  |  |  |
|  | CD72 | CD72 molecule |  |  |  |  |  |  |  |  |  |  |  |  |  |  |  |
|  | CD9 | CD9 molecule |  |  |  |  |  |  |  |  |  |  |  |  |  |  |  |
|  | ELANE | elastase, neutrophil expressed |  |  |  |  |  |  |  |  |  |  |  |  |  |  |  |
|  | FCGR3B | Fc fragment of IgG, low affinity IIIb, receptor (CD16b) |  |  |  |  |  |  |  |  |  |  |  |  |  |  |  |
|  | FLT3 | fms-related tyrosine kinase 3 |  |  |  |  |  |  |  |  |  |  |  |  |  |  |  |
|  | HIST1H2BH | histone cluster 1, H2bh |  |  |  |  |  |  |  |  |  |  |  |  |  |  |  |
|  | HLA-A | major histocompatibility complex, class I, A |  |  |  |  |  |  |  |  |  |  |  |  |  |  |  |
|  | HLA-DMA | major histocompatibility complex, class II, DM alpha |  |  |  |  |  |  |  |  |  |  |  |  |  |  |  |
|  | HLA-DQA1 | major histocompatibility complex, class II, DQ alpha 1 |  |  |  |  |  |  |  |  |  |  |  |  |  |  |  |
|  | HLA-DRB1 | major histocompatibility complex, class II, DR beta 1 |  |  |  |  |  |  |  |  |  |  |  |  |  |  |  |
|  | IGLL1 | immunoglobulin lambda-like polypeptide 1 |  |  |  |  |  |  |  |  |  |  |  |  |  |  |  |
|  | ITK | IL2-inducible T-cell kinase |  |  |  |  |  |  |  |  |  |  |  |  |  |  |  |
|  | LAT | linker for activation of T cells |  |  |  |  |  |  |  |  |  |  |  |  |  |  |  |
|  | MME | membrane metallo-endopeptidase |  |  |  |  |  |  |  |  |  |  |  |  |  |  |  |
|  | NRAS | neuroblastoma RAS viral (v-ras) oncogene homolog |  |  |  |  |  |  |  |  |  |  |  |  |  |  |  |
|  | PIK3AP1 | phosphoinositide-3-kinase adaptor protein 1 |  |  |  |  |  |  |  |  |  |  |  |  |  |  |  |
|  | PPP3CA | protein phosphatase 3 (formerly 2B), catalytic subunit, alpha isoform |  |  |  |  |  |  |  |  |  |  |  |  |  |  |  |
|  | RAG1 | recombination activating gene 1 |  |  |  |  |  |  |  |  |  |  |  |  |  |  |  |
|  | TNFSF13B | tumor necrosis factor (ligand) superfamily, member 13b |  |  |  |  |  |  |  |  |  |  |  |  |  |  |  |
|  |  |  |  |  |  |  |  |  |  |  |  |  |  |  |  |  |  |
| **TaLasso (1/3)** | BLNK | B-cell linker |  |  |  |  |  |  |  |  |  |  |  |  |  |  |  |
|  | CD19 | CD19 molecule |  |  |  |  |  |  |  |  |  |  |  |  |  |  |  |
|  | CD3D | CD3d molecule, delta (CD3-TCR complex) |  |  |  |  |  |  |  |  |  |  |  |  |  |  |  |
|  | CD3G | CD3g molecule, gamma (CD3-TCR complex) |  |  |  |  |  |  |  |  |  |  |  |  |  |  |  |
|  | CD72 | CD72 molecule |  |  |  |  |  |  |  |  |  |  |  |  |  |  |  |
|  | CD9 | CD9 molecule |  |  |  |  |  |  |  |  |  |  |  |  |  |  |  |
|  | FLT3 | fms-related tyrosine kinase 3 |  |  |  |  |  |  |  |  |  |  |  |  |  |  |  |
|  | FOS | FBJ murine osteosarcoma viral oncogene homolog |  |  |  |  |  |  |  |  |  |  |  |  |  |  |  |
|  | HLA-DMA | major histocompatibility complex, class II, DM alpha |  |  |  |  |  |  |  |  |  |  |  |  |  |  |  |
|  | HLA-DQA1 | major histocompatibility complex, class II, DQ alpha 1 |  |  |  |  |  |  |  |  |  |  |  |  |  |  |  |
|  | HLA-DRB1 | major histocompatibility complex, class II, DR beta 1 |  |  |  |  |  |  |  |  |  |  |  |  |  |  |  |
|  | IFITM1 | interferon induced transmembrane protein 1 (9-27) |  |  |  |  |  |  |  |  |  |  |  |  |  |  |  |
|  | IGLL1 | immunoglobulin lambda-like polypeptide 1 |  |  |  |  |  |  |  |  |  |  |  |  |  |  |  |
|  | IL1B | interleukin 1, beta |  |  |  |  |  |  |  |  |  |  |  |  |  |  |  |
|  | ITGA6 | integrin, alpha 6 |  |  |  |  |  |  |  |  |  |  |  |  |  |  |  |
|  | ITK | IL2-inducible T-cell kinase |  |  |  |  |  |  |  |  |  |  |  |  |  |  |  |
|  | LAT | linker for activation of T cells |  |  |  |  |  |  |  |  |  |  |  |  |  |  |  |
|  | LCK | lymphocyte-specific protein tyrosine kinase |  |  |  |  |  |  |  |  |  |  |  |  |  |  |  |
|  | MME | membrane metallo-endopeptidase |  |  |  |  |  |  |  |  |  |  |  |  |  |  |  |
|  | MS4A1 | membrane-spanning 4-domains, subfamily A, member 1 |  |  |  |  |  |  |  |  |  |  |  |  |  |  |  |
|  | NRAS | neuroblastoma RAS viral (v-ras) oncogene homolog |  |  |  |  |  |  |  |  |  |  |  |  |  |  |  |
|  | PIK3AP1 | phosphoinositide-3-kinase adaptor protein 1 |  |  |  |  |  |  |  |  |  |  |  |  |  |  |  |
|  | PPP3CA | protein phosphatase 3 (formerly 2B), catalytic subunit, alpha isoform |  |  |  |  |  |  |  |  |  |  |  |  |  |  |  |
|  | RAG1 | recombination activating gene 1 |  |  |  |  |  |  |  |  |  |  |  |  |  |  |  |
|  | TNFSF13B | tumor necrosis factor (ligand) superfamily, member 13b |  |  |  |  |  |  |  |  |  |  |  |  |  |  |  |
|  |  |  |  |  |  |  |  |  |  |  |  |  |  |  |  |  |  |
| **GenMiR++** | CD2 | CD2 molecule |  |  |  |  |  |  |  |  |  |  |  |  |  |  |  |
|  | CD37 | CD37 molecule |  |  |  |  |  |  |  |  |  |  |  |  |  |  |  |
|  | CD3D | CD3d molecule, delta (CD3-TCR complex) |  |  |  |  |  |  |  |  |  |  |  |  |  |  |  |
|  | CD3E | CD3e molecule, epsilon (CD3-TCR complex) |  |  |  |  |  |  |  |  |  |  |  |  |  |  |  |
|  | CD3G | CD3g molecule, gamma (CD3-TCR complex) |  |  |  |  |  |  |  |  |  |  |  |  |  |  |  |
|  | CD7 | CD7 molecule |  |  |  |  |  |  |  |  |  |  |  |  |  |  |  |
|  | FOS | FBJ murine osteosarcoma viral oncogene homolog |  |  |  |  |  |  |  |  |  |  |  |  |  |  |  |
|  | ITK | IL2-inducible T-cell kinase |  |  |  |  |  |  |  |  |  |  |  |  |  |  |  |
|  | KIT | v-kit Hardy-Zuckerman 4 feline sarcoma viral oncogene homolog |  |  |  |  |  |  |  |  |  |  |  |  |  |  |  |
|  | LAT | linker for activation of T cells |  |  |  |  |  |  |  |  |  |  |  |  |  |  |  |
|  | LCK | lymphocyte-specific protein tyrosine kinase |  |  |  |  |  |  |  |  |  |  |  |  |  |  |  |
|  | PRKCQ | protein kinase C, theta |  |  |  |  |  |  |  |  |  |  |  |  |  |  |  |
|  | RASGRP1 | RAS guanyl releasing protein 1 (calcium and DAG-regulated) |  |  |  |  |  |  |  |  |  |  |  |  |  |  |  |
|  | ZAP70 | zeta-chain (TCR) associated protein kinase 70kDa |  |  |  |  |  |  |  |  |  |  |  |  |  |  |  |
