# Supplementary material for: Quantification of miRNA-mRNA Interactions
Source: PLoS One. 2012 Feb 14;7(2):e30766. doi: 10.1371/journal.pone.0030766 (PMC3279346; doi:10.1371/journal.pone.0030766)
Supplement: Text S3 — Reference articles for the experimentally-validated targets on the top 500 interactions for MCC dataset. (DOC) [file pone.0030766.s005.doc]

## Reference articles for the experimentally validated targets on the top 500 interactions

In this text S3 the citations for each experimentally validated targets associated to the experimental biology behind MCC shown in the table 3 of the article are included. The full reference of each citation is included after the table.

**Table 1.** **Predicted experimentally validated targets and the cancer to which they have been related in the literature: results for MCC dataset.**

| **Interactions** | | **Database** | | | **Method** | | | | **Associated to** | | | | | | | | |
| --- | --- | --- | --- | --- | --- | --- | --- | --- | --- | --- | --- | --- | --- | --- | --- | --- | --- |
| ***Gene*** | ***miRNA*** | ***TaRBase*** | ***miRecords*** | ***miRWalk*** | ***TaLasso (1/5)*** | ***TaLasso (1/10)*** | ***GenMiR++*** | ***Correlation*** | ***OV*** | ***BC*** | ***EC*** | ***CC*** | ***LC*** | ***BrC*** | ***NC*** | ***PC*** | ***GC*** |
| EEF1A2 | let-7f |  | **X** | **X** | **X** | **X** |  |  | [1] |  |  |  |  |  |  |  |  |
| FSCN1 | miR-133a |  |  | **X** | **X** | **X** |  |  |  | [2] | [3] |  |  |  |  |  |  |
| FSCN1 | miR-145 |  |  | **X** | **X** | **X** |  |  |  | [2] | [3]-[4] | [5] |  |  |  |  |  |
| BRAF | miR-192 |  |  | **X** |  | **X** |  |  |  |  |  | [6] |  |  |  |  |  |
| CCND1 | miR-194 |  |  | **X** |  | **X** |  |  |  |  |  |  | [7] |  |  |  |  |
| HOXD10 | miR-200c |  |  | **X** |  | **X** |  |  |  | [8] |  | [8] | [8] | [8] |  |  |  |
| CDKN2A | miR-99a |  |  | **X** |  | **X** |  |  |  |  |  |  | [9] |  |  |  |  |
| TFF1 | let-7f |  | **X** | **X** | **X** |  |  |  | [1] |  |  |  |  |  |  |  |  |
| CEACAM5 | miR-143 / 145 |  |  | **X** | **X** |  |  |  |  |  |  | [10] |  |  |  |  |  |
| KRT14 | miR-143 |  |  | **X** | **X** |  |  |  |  |  | [11] |  |  |  |  |  |  |
| KRT7 | miR-145 / 195 |  |  | **X** | **X** |  |  |  |  | [12] |  |  |  |  |  |  |  |
| INS | miR-27a |  |  | **X** | **X** |  |  |  | [13] |  |  |  |  | [13] |  |  |  |
| IGFBP6 | miR-27a |  |  | **X** | **X** |  |  |  | [13] |  |  |  |  | [13] |  |  |  |
| PIGR | miR-125b |  | **X** | **X** |  |  | **X** |  |  |  |  |  |  |  |  | [14] |  |
| BRAF | miR-145 |  |  | **X** |  |  | **X** |  |  |  |  | [6] |  |  |  |  |  |
| PLK1 | miR-100 |  |  | **X** |  |  |  | **X** |  |  |  |  |  |  | [15] |  |  |
| BAK1 | miR-125b |  | **X** |  |  |  |  | **X** |  |  |  |  |  | [16] |  |  |  |
| E2F1 | miR-195 |  |  | **X** |  |  |  | **X** |  |  |  |  |  |  |  |  | [17] |
| TWIST1 | miR-141 |  |  | **X** |  |  |  | **X** |  |  |  |  |  | [18] |  |  |  |
| TWIST1 | miR-200c |  |  | **X** |  |  |  | **X** |  | [10] |  |  |  | [18]-[19] |  |  |  |

CLL: Chronic Lymphoblastic Leukaemia, ALL: Acute Lymphoblastic Leukaemia, AML: Acute Myeloid Leukaemia, IC: Immunce Cells, IR: Immune Response, HSC: Haematopoietic SC.

The experimentally validated targets included in the top 500 targets predicted were selected and their literature references included on TaRBase, miRecords and miRWalk were analyzed in search of biological relevancy. In the table only those interactions with a literature reference related with MCC environment have been included. This was made for the predictions of TaLasso, GenMiR++ and Pearson Correlation.

**Corresponding references**

1.     Dahiya N, Sherman-Baust CA, Wang TL, Davidson B, Shih I, et al. (2008) MicroRNA expression and identification of putative miRNA targets in ovarian cancer PLoS One 3: e2436.

2.     Baffa R, Fassan M, Volinia S, O'Hara B, Liu CG, et al. (2009) MicroRNA expression profiling of human metastatic cancers identifies cancer gene targets J Pathol 219: 214-221.

3.     Shi XB, Xue L, Yang J, Ma AH, Zhao J, et al. (2007) An androgen-regulated miRNA suppresses Bak1 expression and induces androgen-independent growth of prostate cancer cells Proc Natl Acad Sci U S A 104: 19983-19988.

4.     Chiyomaru T, Enokida H, Tatarano S, Kawahara K, Uchida Y, et al. (2010) miR-145 and miR-133a function as tumour suppressors and directly regulate FSCN1 expression in bladder cancer Br J Cancer 102: 883-891.

5.     Nagayama K, Kohno T, Sato M, Arai Y, Minna JD, et al. (2007) Homozygous deletion scanning of the lung cancer genome at a 100-kb resolution Genes Chromosomes Cancer 46: 1000-1010.

6.     Shi W, Alajez NM, Bastianutto C, Hui AB, Mocanu JD, et al. (2010) Significance of Plk1 regulation by miR-100 in human nasopharyngeal cancer Int J Cancer 126: 2036-2048.

7.     Kano M, Seki N, Kikkawa N, Fujimura L, Hoshino I, et al. (2010) miR-145, miR-133a and miR-133b: Tumor suppressive miRNAs target FSCN1 in esophageal squamous cell carcinoma Int J Cancer .

8.     Slaby O, Svoboda M, Fabian P, Smerdova T, Knoflickova D, et al. (2007) Altered expression of miR-21, miR-31, miR-143 and miR-145 is related to clinicopathologic features of colorectal cancer Oncology 72: 397-402.

9.     Zhou M, Liu Z, Zhao Y, Ding Y, Liu H, et al. (2010) MicroRNA-125b confers the resistance of breast cancer cells to paclitaxel through suppression of pro-apoptotic bcl-2 antagonist killer 1 (Bak1) expression J Biol Chem 285: 21496-21507.

10.     Wu BL, Xu LY, Du ZP, Liao LD, Zhang HF, et al. (2011) MiRNA profile in esophageal squamous cell carcinoma: Downregulation of miR-143 and miR-145 World J Gastroenterol 17: 79-88.

11.     Dijckmeester WA, Wijnhoven BP, Watson DI, Leong MP, Michael MZ, et al. (2009) MicroRNA-143 and -205 expression in neosquamous esophageal epithelium following argon plasma ablation of barrett's esophagus J Gastrointest Surg 13: 846-853.

12.     Guo J, Miao Y, Xiao B, Huan R, Jiang Z, et al. (2009) Differential expression of microRNA species in human gastric cancer versus non-tumorous tissues J Gastroenterol Hepatol 24: 652-657.

13.     Gregersen LH, Jacobsen AB, Frankel LB, Wen J, Krogh A, et al. (2010) MicroRNA-145 targets YES and STAT1 in colon cancer cells PLoS One 5: e8836.

14.     Ichimi T, Enokida H, Okuno Y, Kunimoto R, Chiyomaru T, et al. (2009) Identification of novel microRNA targets based on microRNA signatures in bladder cancer Int J Cancer 125: 345-352.

15.     Neves R, Scheel C, Weinhold S, Honisch E, Iwaniuk KM, et al. (2010) Role of DNA methylation in miR-200c/141 cluster silencing in invasive breast cancer cells BMC Res Notes 3: 219.

16.     Yantiss RK, Goodarzi M, Zhou XK, Rennert H, Pirog EC, et al. (2009) Clinical, pathologic, and molecular features of early-onset colorectal carcinoma Am J Surg Pathol 33: 572-582.

17.     Kontorovich T, Levy A, Korostishevsky M, Nir U, Friedman E. (2010) Single nucleotide polymorphisms in miRNA binding sites and miRNA genes as breast/ovarian cancer risk modifiers in jewish high-risk women Int J Cancer 127: 589-597.

18.     Wiklund ED, Bramsen JB, Hulf T, Dyrskjot L, Ramanathan R, et al. (2011) Coordinated epigenetic repression of the miR-200 family and miR-205 in invasive bladder cancer Int J Cancer 128: 1327-1334.

19.     Ohlsson Teague EM, Van der Hoek KH, Van der Hoek MB, Perry N, Wagaarachchi P, et al. (2009) MicroRNA-regulated pathways associated with endometriosis Mol Endocrinol 23: 265-275.
