# Supplementary material for: Quantification of miRNA-mRNA Interactions
Source: PLoS One. 2012 Feb 14;7(2):e30766. doi: 10.1371/journal.pone.0030766 (PMC3279346; doi:10.1371/journal.pone.0030766)
Supplement: Text S4 — Reference articles for the experimentally-validated targets on the top 500 interactions for LDS dataset. (DOC) [file pone.0030766.s006.doc]

## Reference articles for the experimentally validated targets on the top 500 interactions

In this text S4 the citations for each experimentally validated targets associated to the experimental biology behind LDS shown in the tables 3 and 4 of the article are included. The full reference of each citation is included after the table.

**Table 1.** **Predicted experimentally validated targets and the cancer to which they have been related in the literature: results for LDS dataset.**

| **Interactions** | | **Exp. Val. Database** | | | **Method** | | | | **Associated to** | | | |
| --- | --- | --- | --- | --- | --- | --- | --- | --- | --- | --- | --- | --- |
| ***gene*** | ***miRNA*** | ***TaRBase*** | ***miRecords*** | ***miRWalk*** | ***TaLasso (1/2)*** | ***TaLasso (1/3)*** | ***GenMiR++*** | ***Correlation*** | ***CLL*** | ***ALL*** | ***AML*** | ***IC, IR, HSC*** |
| GPR160 | miR-125b |  | **X** | **X** |  | **X** |  |  |  |  |  | [1] |
| FLT3 | miR-148a |  |  | **X** | **X** | **X** |  |  |  |  | [2] |  |
| BAALC | miR-148a |  |  | **X** | **X** | **X** |  |  |  |  | [2]-[3] |  |
| TCL1A | miR-15a, miR-16 |  |  | **X** |  | **X** |  |  | [4]-[5]-[6] |  |  |  |
| TCL1A | miR-181b, miR-29b |  |  | **X** |  | **X** |  |  | [6]-[7] |  |  |  |
| TCL1A | miR-34b |  |  | **X** |  | **X** |  |  | [8] |  |  |  |
| CD19 | miR-29c |  |  | **X** |  | **X** |  |  | [9] | [9] |  |  |
| HLA-DPA1 | miR-181a, miR-223 |  |  | **X** |  | **X** |  |  |  |  |  | [10] |
| KIT | miR-221, miR-222 | **X** | **X** | **X** |  |  | **X** |  |  |  | [11]- [12] | [13] |
| ZAP70 | miR-155 |  |  | **X** |  |  | **X** |  | [14]-[15] |  |  |  |
| ZAP70 | miR-16 |  |  | **X** |  |  | **X** |  | [5]-[15] |  |  |  |
| ZAP70 | miR-181a |  |  | **X** |  |  | **X** |  | [14]-[15]-[16] |  |  |  |
| PLK2 | miR-126 |  |  | **X** |  |  | **X** |  |  | [17] |  |  |
| ETS1 | miR-155 |  |  | **X** |  |  | **X** |  |  |  |  | [18] |
| HOXA9 | miR-126 |  |  | **X** |  |  | **X** |  |  |  |  | [19] |
| CCNA1 | let-7b | **X** |  | **X** |  | **X** | **X** | **X** |  |  |  |  |

CLL: Chronic Lymphoblastic Leukaemia, ALL: Acute Lymphoblastic Leukaemia, AML: Acute Myeloid Leukaemia, IC: Immunce Cells, IR: Immune Response, HSC: Haematopoietic SC.

The experimentally validated targets included in the top 500 targets predicted were selected and their literature references included on TaRBase, miRecords and miRWalk were analyzed in search of biological relevancy. In the table only those interactions with a literature reference related with LDS environment have been included. This was made for the predictions of TaLasso, GenMiR++ and Pearson Correlation.

**Corresponding references**

1.     Lee JE, Hong EJ, Nam HY, Kim JW, Han BG, et al. (2011) MicroRNA signatures associated with immortalization of EBV-transformed lymphoblastoid cell lines and their clinical traits Cell Prolif 44: 59-66.

2.     Langer C, Radmacher MD, Ruppert AS, Whitman SP, Paschka P, et al. (2008) High BAALC expression associates with other molecular prognostic markers, poor outcome, and a distinct gene-expression signature in cytogenetically normal patients younger than 60 years with acute myeloid leukemia: A cancer and leukemia group B (CALGB) study Blood 111: 5371-5379.

3.     Schwind S, Marcucci G, Maharry K, Radmacher MD, Mrozek K, et al. (2010) BAALC and ERG expression levels are associated with outcome and distinct gene and microRNA expression profiles in older patients with de novo cytogenetically normal acute myeloid leukemia: A cancer and leukemia group B study Blood 116: 5660-5669.

4.     Dong C, Ji M, Ji C. (2009) microRNAs and their potential target genes in leukemia pathogenesis Cancer Biol Ther 8: 200-205.

5.     Calin GA, Pekarsky Y, Croce CM. (2007) The role of microRNA and other non-coding RNA in the pathogenesis of chronic lymphocytic leukemia Best Pract Res Clin Haematol 20: 425-437.

6.     Pekarsky Y, Calin GA, Aqeilan R. (2005) Chronic lymphocytic leukemia: Molecular genetics and animal models Curr Top Microbiol Immunol 294: 51-70.

7.     Pekarsky Y, Santanam U, Cimmino A, Palamarchuk A, Efanov A, et al. (2006) Tcl1 expression in chronic lymphocytic leukemia is regulated by miR-29 and miR-181 Cancer Res 66: 11590-11593.

8.     Cardinaud B, Moreilhon C, Marcet B, Robbe-Sermesant K, LeBrigand K, et al. (2009) miR-34b/miR-34c: A regulator of TCL1 expression in 11q- chronic lymphocytic leukaemia? Leukemia 23: 2174-2177.

9.     Zanette DL, Rivadavia F, Molfetta GA, Barbuzano FG, Proto-Siqueira R, et al. (2007) miRNA expression profiles in chronic lymphocytic and acute lymphocytic leukemia Braz J Med Biol Res 40: 1435-1440.

10.     Pedersen I, David M. (2008) MicroRNAs in the immune response Cytokine 43: 391-394.

11.     Felli N, Fontana L, Pelosi E, Botta R, Bonci D, et al. (2005) MicroRNAs 221 and 222 inhibit normal erythropoiesis and erythroleukemic cell growth via kit receptor down-modulation Proc Natl Acad Sci U S A 102: 18081-18086.

12.     Brioschi M, Fischer J, Cairoli R, Rossetti S, Pezzetti L, et al. (2010) Down-regulation of microRNAs 222/221 in acute myelogenous leukemia with deranged core-binding factor subunits Neoplasia 12: 866-876.

13.     Kuipers H, Schnorfeil FM, Brocker T. (2010) Differentially expressed microRNAs regulate plasmacytoid vs. conventional dendritic cell development Mol Immunol 48: 333-340.

14.     Visone R, Rassenti LZ, Veronese A, Taccioli C, Costinean S, et al. (2009) Karyotype-specific microRNA signature in chronic lymphocytic leukemia Blood 114: 3872-3879.

15.     Wang M, Tan LP, Dijkstra MK, van Lom K, Robertus JL, et al. (2008) miRNA analysis in B-cell chronic lymphocytic leukaemia: Proliferation centres characterized by low miR-150 and high BIC/miR-155 expression J Pathol 215: 13-20.

16.     Zhou JY, Ma WL, Fei J, Ding DP, Shi R, et al. (2006) Effects of microRNA miR-181a on gene expression profiles of K562 cells] Nan Fang Yi Ke Da Xue Xue Bao 26: 606-609.

17.     Li Z, Lu J, Sun M, Mi S, Zhang H, et al. (2008) Distinct microRNA expression profiles in acute myeloid leukemia with common translocations Proc Natl Acad Sci U S A 105: 15535-15540.

18.     Romania P, Lulli V, Pelosi E, Biffoni M, Peschle C, et al. (2008) MicroRNA 155 modulates megakaryopoiesis at progenitor and precursor level by targeting ets-1 and Meis1 transcription factors Br J Haematol 143: 570-580.

19.     Shen W, Hu Y, Uttarwar L, Passegue E, Largman C. (2008) MicroRNA-126 regulates HOXA9 by binding to the homeobox. Mol Cell Biol 28: 4609-19.
