# Supplementary material for: Quantification of miRNA-mRNA Interactions
Source: PLoS One. 2012 Feb 14;7(2):e30766. doi: 10.1371/journal.pone.0030766 (PMC3279346; doi:10.1371/journal.pone.0030766)
Supplement: Text S5 — Implementation guidelines for the software and the web-page for running TaLasso. (DOC) [file pone.0030766.s007.doc]

## Implementation

## TaLasso: the web-based tool

The TaLasso web site is an easy tool where only a few steps are needed to get the interaction scoring results provided by the TaLasso algorithm (figure 4):


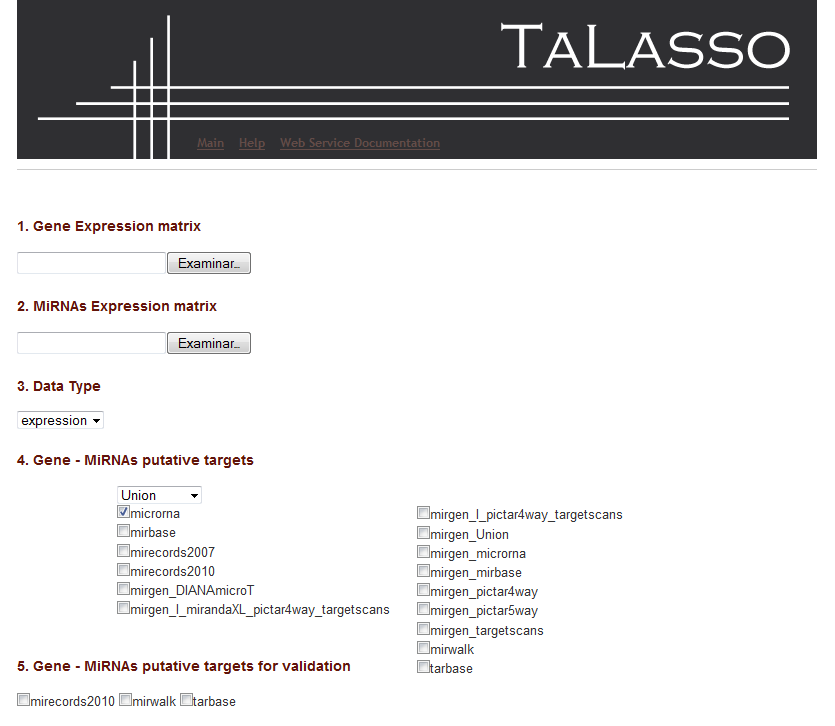


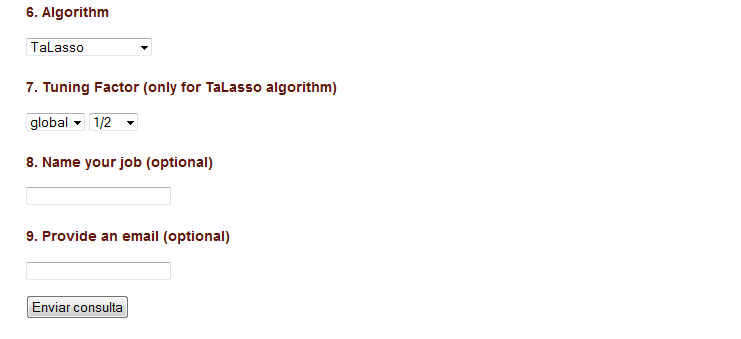


**Fig. 4**. View of the TaLasso web-site.

1. The first step is uploading the mRNA and miRNA expression data. Both expression data must be tab delimited and must have the sample names in the first row and ensemble gene IDs of miRBase mature miRNA names in the first column. The uploading is made by selecting the text files in points 1 and 2 of figure 4.
2. Since miRNA data can be ΔΔCt or expression data, this option could be selected in point 3.
3. Next, the set of interactions must be selected from the list of the different putative targets databases provided (step 4). It must be also determined whether the putative interactions will be the union or the intersection of the selected databases.
4. The last step before the selection of the method for scoring is to choose the experimentally validated database for the statistical quality analysis (only in case union option is selected and if the corresponding experimentally validated database has been selected on point 4).
5. Finally, users have to select the method for interaction scoring: Pearson Correlation, GenMiR++ or TaLasso (step 6). For this last method, the tuning parameter has to be determined: global (a general one for all the mRNAs) or local (one for each mRNA) and its value (step 7).
6. As optional choice, users can name its work to have a reference once the results are obtained. They can also indicate their e-mail address to be notified when the analysis will be finished.

Once the algorithm has finished, the results will be placed in the web-page (figure 5). Its structure it is simple:

1. Interactions are placed sorted in a table in descendent order by score.
2. It is possible to click in the image placed next to each gene name to explore all the miRNAs that regulate that mRNA. In the same way, it is possible to click in the image placed next to each miRNA name to explore all the genes regulated by that miRNA. Finally, clicking on gene names opens the ensemble reference page of the gene.
3. The right columns indicate if the interaction is included in miRecords, miRWalk or TaRBase databases.
4. The p-values of the multiple linear regression of the results are placed in the column pValue.
5. The figure on the top of the results page corresponds to the hypergeometric test on the experimentally validated targets selected and a hit distribution map of the interactions. The images become bigger by clicking on them.

On the bottom of the results page, a button called “Functional enrichment” has been included to allow users to get a functional enrichment analysis provided by GeneCodis tool of the top 200 genes within the top-ranked interactions.


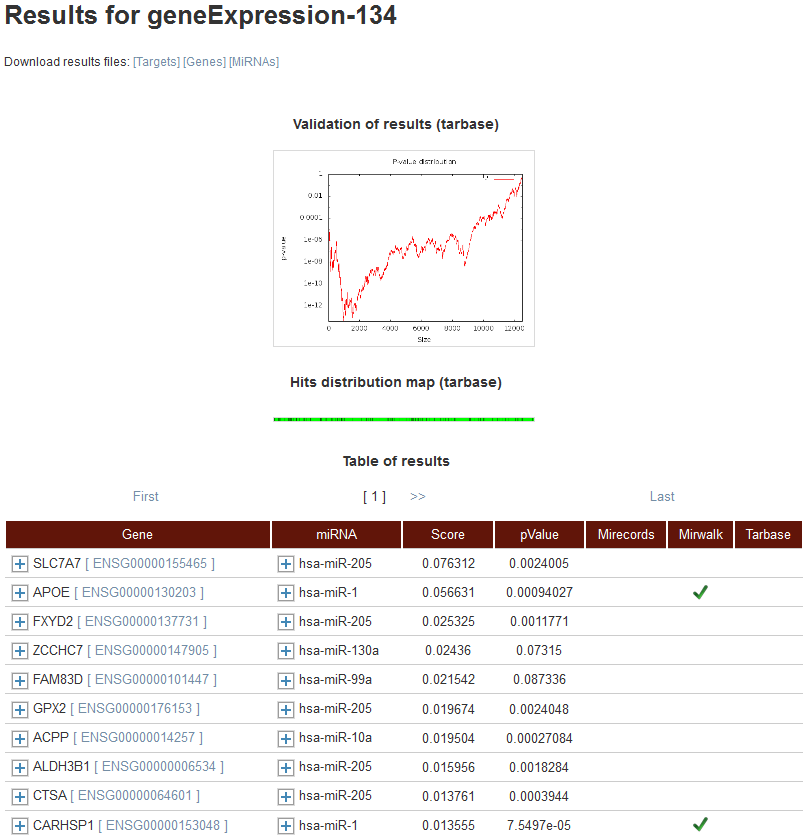


**Fig. 5**. View of the TaLasso web-site.

## Running TaLasso in Matlab and R

## The structure of folders in TaLasso

TaLasso can be run using Matlab or R. Both R and Matlab share the folder structure as shown in figure 2: *code*, *data*, *library* and *results*. These folders are stored in *TaLasso* and *TaLasso_R* folders for Matlab and R softwares respectively.

- The experimental data must be included within a folder in the “*data*” folder. The name of the folder is used to describe the particular experimental data.
- The folder *library* contains the putative targets databases, and gene and miRNA names.
- The folder *code* contains all the code functions (*.m or *.R files). ‘main.m’ or ‘main.R’ are the only code to be executed to run TaLasso algorithm.
- Once TaLasso is run, the algorithm will save the results on the folder *result*. TaLasso assign to each solution a particular name indicating: 1) the value of *Gamma*, 2) data normalization method (*M* = median), 3) the type of tuning parameter used (*global* or *local*), 4) the name of the experimental data (i.e. *MCC* or *LDS*) and 5) the tuning parameters’ factor used.


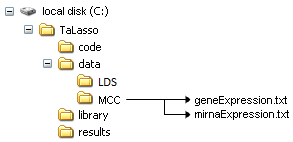


**Fig. 6**. Folder structure for TaLasso

- 1. **The structure of expression data**

MRNA and miRNA expressions data for each experiment must be a tab delimited text file named geneExpression.txt and mirnaExpression.txt respectively. The first row on each file must contain sample names (with no free spaces) and the first column must contain miRNA (miRBase ID) or gene (Ensembl ID) names. See folders LDS and MCC facilitated on data for examples. The miRNA expression data of LDS and MCC experiments corresponds to expression values.

- 1. **Running TaLasso**

The only code to be executed to run TaLasso in both softwares is the file ‘main.m’ or ‘main.R’. However, ‘Rcplex’ package must be installed before R code is run. This installation is a little bit involved. First of all there must be a running copy of Cplex. Afterwards, the user must also download and install the ‘cplex’ software. Once ‘cplex’ is installed, the ‘Rcplex’ package must be compiled by following the instructions specified on <http://CRAN.R-project.org/package=Rcplex>.

The code included on ‘main.m’ is shown in the following paragraph (is almost identical for main.R). Each of the input parameters are:

1. *Directory* = is the directory where the folder TaLasso has been placed.
2. *Data* = is the folder name where the files ‘geneExpression.txt’and ‘mirnaExpression.txt’ for the experiment to analyze are stored.
3. *Data_Type* = refers to miRNA data: ‘expression’ in case miRNA data corresponds to expression levels and ‘DDCt’ in case miRNA data refers to ΔΔCt data (qPCR experiment).
4. *databases_num* = is a vector of numbers referring to the databases chosen and from the list on ‘main.m’.
5. *option* = a variable indicating whether the putative targets matrix must be the union (‘union’) or the intersection (‘intersect’) of those chosen on databases_num.
6. *TuningFactor_Type* = indicates if TaLasso must use ‘global’ or ‘local’ tuning parameters
7. *TuningFactor* = is the value of the tuning factor to use.

% main script for calling TaLasso

directory = 'C:\TaLasso';

%-----------------------------------%

% experimental data to be processed %

%-----------------------------------%

Data = 'MCC';

Data_Type = 'expression';

%------------------%

% select databases %

%------------------%

% 1: 'microrna'

% 2: 'mirbase'

% 3: 'mirecords2007'

% 4: 'mirecords2010'

% 5: 'mirgen_DIANAmicroT'

% 6: 'mirgen_I_mirandaXL_pictar4way_targetscans'

% 7: 'mirgen_I_pictar4way_targetscans'

% 8: 'mirgen_Union'

% 9: 'mirgen_microrna'

% 10: 'mirgen_mirbase'

% 11: 'mirgen_pictar4way'

% 12: 'mirgen_pictar5way'

% 13: 'mirgen_targetscans'

% 14: 'mirwalk'

% 15: 'tarbase'

databases_num = [ 1 , 2 , 4 , 8 , 14 , 15 ];

option = 'union';

%------------------------------%

% chose parameters for TaLasso %

%------------------------------%

TuningFactor_Type = 'global';

TuningFactor = 1/100;

%---------------%

% solve TaLasso %

%---------------%

main_TaLasso( directory , Data , Data_Type , databases_num , option , TuningFactor_Type , TuningFactor );

The statistical significance of the results can be obtained by running the function *significance_beta* (or its equivalent in R):

[ pval , F , R2 ] = significance_beta( solution , offset , X , Z , C , info );
