# Supplementary material for: Quantification of miRNA-mRNA Interactions
Source: PLoS One. 2012 Feb 14;7(2):e30766. doi: 10.1371/journal.pone.0030766 (PMC3279346; doi:10.1371/journal.pone.0030766)
Supplement: Text S6 — Statistical significance of the results obtained by TaLasso. (DOC) [file pone.0030766.s008.doc]

## Statistical significance of the solutions

To date, there is no method that provides reliable confidence intervals for regularized and constrained Least Squares. In Talasso, an *approximation* to the statistical significance of the solution is estimated by using multiple linear regression statistics. Assuming as null hypothesis that the *β_jk_* values are zero, Talasso estimates the single tailed p-values provided by a linear model as an approximation to the p-values of the constrained Lasso regression. The procedure is as follows:

- 1. Solve TaLasso for all mRNAs.
  2. For each mRNA, select the top (1...n-1) miRNA regulators ordered by the *β_jk_* value computed by TaLasso and apply sequentially n-1 linear models to that mRNA and its (1...n-1) miRNA regulators where n is the number of samples
  3. From these n-1 linear models, select the p regulators that provide the minimum F-value
  4. Finally, using this linear model with p regulators, assign to each of the interactions the corresponding p-values of the linear model.

These steps are automatically run in the web server. This algorithm is implemented in the function called *significance_beta*, in both R and Matlab codes.
